# Dynamics of botnet propagation model in complex networks considering hybrid method for botnet detection

**DOI:** 10.1371/journal.pone.0345157

**Published:** 2026-06-09

**Authors:** Mahdieh Maazalahi, Soodeh Hosseini

**Affiliations:** Department of Computer Science, Faculty of Mathematics and Computer, Shahid Bahonar University of Kerman, Kerman, Iran; Federal University of Minas Gerais: Universidade Federal de Minas Gerais, BRAZIL

## Abstract

In this paper, a dynamic epidemic model of botnet attack propagation in scale-free networks is introduced based on the epidemic model. The proposed attack propagation model is based on the Susceptible-Exposure-Infected-Improved-Vaccinated-Recovery (SEIRVS) epidemic model. Here, an Intrusion Detection System (IDS) for botnet attack detection is also presented. This method is based on a combination of machine learning and metaheuristic algorithms, the Golden Ratio Optimization (GRO) algorithm, Bat Algorithm (BA), and K-Nearest Neighbor (KNN) algorithms named (GRO-BA-K-NN), which includes three steps: 1) preprocessing, 2) GRO feature selection 3) attack detection using BA-K-NN. The proposed IDS, using the three datasets BOT-IOT, UNSW-NB15, and NLS-KDD, and the dynamic behavior of the proposed model, is evaluated using the metric of the initial production ratio; evaluating the dynamic behavior of the model can be used to predict whether the infection spreads or stops. The evaluation results show that the epidemic model reduces the density of infected nodes and stops the spread of infection compared to other existing models. The simulation results show that the proposed IDS was able to detect attacks with accuracy (0.938, 0.931, and 0.928) and also reduced the false negative and false positive rates.

## 1. Introduction

Real-world networks such as social networks, the Internet, and biological networks are a type of scale-independent network. One of the most important features of these networks is that they follow a power law. In a power law, a small number of nodes have many connections, and most nodes have fewer connections [1–2].

The Internet plays a vital role in everyday life, supporting social interactions and raising privacy concerns. Botnet attacks are a security threat that has seen tens of thousands of simultaneous attacks worldwide. Among the most dangerous attacks, the Mirai attack in 2016 targeted Dyn DNS and caused widespread outages [3]. The methods used by cybercriminals to exploit technology for malicious purposes are expanding and evolving. In 2023, there were more than 12.3 billion devices connected worldwide, and this number is expected to exceed 25 billion by 2030 [4]. Botnet attacks are a security threat that has seen tens of thousands of simultaneous attacks worldwide. This shows how important it is to have reliable and secure online services. Botnet attacks are a major threat to online activities. These attacks are a network of infected computers, called bots, controlled by cybercriminals. These bots are suitable for large-scale cyberattacks aimed at taking down servers [5].

In the past few years, various methods have been proposed to detect botnet attacks. Some methods include new hybrid algorithms for detecting attacks, and some other methods are new methods to reduce the rate of malware propagation. An effective approach to detecting botnet attacks is to use machine learning (ML) algorithms and metaheuristic (MH) algorithms. ML and MH algorithms detect attacks on high-dimensional data sets to distinguish malicious and normal traffic from each other [6].

**Fig 1:**
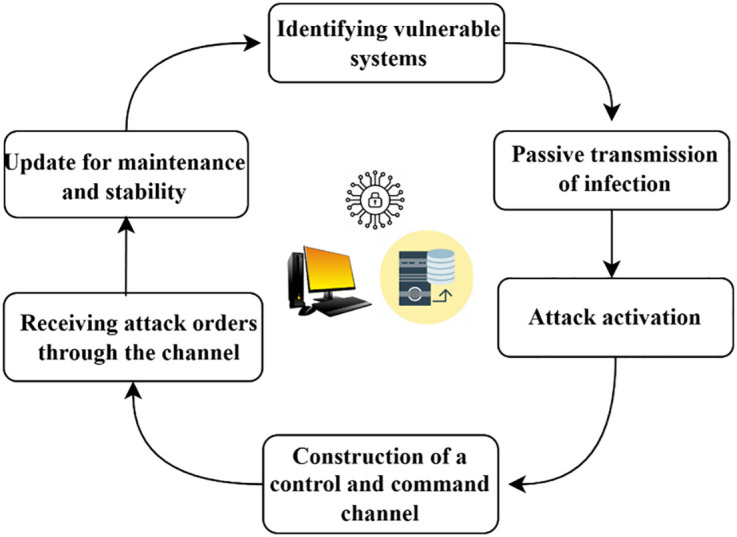
The stages of botnet attacks.

Detecting botnet attacks in a scale-free network is important because of the power law of hubs, which is why attackers use nodes with the highest degree and disrupt them to control the network. The factors needed to secure these networks are: 1) Monitoring high-degree nodes because they are critical. 2) Using anomaly detection algorithms such as machine learning and metaheuristics to identify infections, focusing on network traffic, especially the behavior of hubs. 3) Analyzing network traffic to find signs of command and control (C&C) communications in botnets. Mathematical modeling is a valuable tool for discovering the mechanisms behind attack propagation and the effects of attack propagation. Metaheuristic algorithms are high-level, flexible optimization methods designed to find near-optimal solutions to complex problems [7]. The performance of each algorithm is improved by using a hybrid approach. Botnet attacks are a type of cyber threat that involves a network of infected computers called bots that are controlled by an attacker. The general behavior of botnet attacks includes the following: 1) Targeting and infecting: First, vulnerable systems are identified and infected, turning them into bots. 2) Command and control (C&C): Bots receive commands from an attacker using C&C servers and execute them. 3) Attack execution: Bots execute attacks using attack instructions sent to the bots through a command-and-control channel. Here, a propagation model called SEIRVS is presented to illustrate the dynamics of a botnet attack in a heterogeneous scale-free network. Fig 1 shows the stages of botnet attacks [8].

### 1.1. Motivation and contribution

In this paper, a new method for the epidemic model of botnet attack propagation in heterogeneous networks is presented. In addition, an intrusion detection system (IDS) combining metaheuristic and machine learning algorithms called Golden Ratio Optimization (GRO), Bat Algorithm (BA), and K-Nearest Neighbor (K-NN) algorithm, abbreviated as GRO-BA-K-NN, is presented. The advantages of the epidemic model and the proposed IDS are:

- Based on the disease propagation model, a new dynamic model for the propagation of botnet attacks, called SEIRVS, is introduced. In this model, two modes of recovery and vaccination are considered. To demonstrate the effectiveness of this model, 1) the effects of various parameters, such as recovery, vaccination, contamination, and vulnerability, on reducing the spread of attacks are studied. 2) We investigate the dynamic behaviors of the model and obtain the equilibrium point without attacks. 3) The basic reproduction ratio criterion is calculated to control the spread of botnet attacks in the network.
- Here, a hybrid intrusion detection system is also presented to detect botnet attacks. In this method, after preprocessing the dataset using the GRO algorithm, features with the lowest fitness value are selected. This algorithm selects a subset of features that are neither too high nor too low to create a balance. This prevents overfitting and selects a more robust set of features. For attack detection, the BA algorithm is used for training and the K-NN algorithm is used for classifying attacks. • The proposed method for detecting botnet attacks is evaluated using three datasets: BOT-IOT, UNSW-NB15, and NLS-KDD, and various metrics.

The remainder of the paper is organized as follows. The related works section examines attack detection by utilizing machine learning algorithms and evolutionary methods and malware epidemic models. The proposed model section presents a new model for botnet attack propagation and an intrusion detection system for identifying attacks. The dynamic analysis of the model section analyzes the dynamics of the SEIRVS model and presents the equilibrium stability method. the experiments and simulations section examines, describes, and covers performance metrics such as accuracy, Precision, Recall, F-measure, specificity, and G-mean from the numerical simulations of the model and the intrusion detection system. The discussion section provides an analysis that, by interpreting prior research, highlights the main contributions of the study, and finally, Conclusions and Future Works Section, presents the conclusions and future work.

## 2. Related work

In the last few years, a lot of research has been done on modeling the spread of malware and various attacks in heterogeneous networks and on various combined and single IDSs for detecting attacks. In the following, the work done in each of these areas is reviewed separately.

### 2.1. Epidemic malware model

This section describes the malware epidemic models of the last few years and compares them in Table 1.

**Table 1:** Investigation of pollution diffusion models in heterogeneous systems.

Authors	Method	Advantage	Disadvantage	Environment
G. Liu *et al.* (2025) [9]	Used the VCISQ model to spread malware	They perform well when faced with changes in parameters.	Ignoring hostile capabilities.	Wireless Rechargeable Sensor Network (WRSNs)
D. C. Padilla *et al.* (2025) [10]	Used the SERDUX-MARCIM model spread of cyberattacks on infrastructure	Suitable for real-world scenarios	1)Incompatibility of the method with different types of cyberthreats. 2)Failure to consider changes in attacker behavior over time	
L. Q. Sánchez *et al.* (2025) [11]	Used SEIRS-NIMFA malware propagation	Identify nodes to prioritize security tasks	1)Failure to implement the model in large networks. 2)This model is unsuitable for networks with variable sizes.	Internet of Things (IOT)
G. Wu *et al*. (2024) [12]	Used SIHQR for worm propagation	This model can reduce the Hopf bifurcation.	–	Industrial Internet of Things (IIOT)
A. M. Rey (2024) [13]	Used the SS_P_LL_I_AA_I_DQ model to spread malware	1)Taking key actions to contain the spread using the reproduction number analysis. 2) Having a global nature, where the variables represent the density of the epidemiological component.	1)Failure to consider the characteristics of each WSN device. 2) Failure to consider epidemiological coefficients of malware propagation models. 3) High complexity of the analysis model reduces	Wireless Sensor Network (WSN)
E. Asadi and S. Hosseini (2024) [14]	Used the SEIRD model to spread malware	Combining epidemic propagation and clustering methods	–	scale-free networks
A. Chernikova *et al.* (2023) [15]	Used the SII_D_R model to spread malware	Suitable for simulating attacks in heterogenous network	–	

G. Liu *et al.* [9] proposed a real-world propagation model, which includes nodes V (Vulnerable), C (Carrier), I (Infectious), S (Secured), and Q (Quarantined). This model considers the critical features of node density, time characteristics, and fading channels, and also uses an optimal control method to reduce the control costs. Proximal Policy Optimization (PPO) and Multi-Agent Proximal Policy Optimization (MAPPO) are used to formulate the decision process. The evaluation results show that this method can control the costs and convergence.

D. C. Padilla *et al.* [10] proposed the SERDUX-MARCIM method, which is based on the propagation of cyber attacks on maritime infrastructure. This model is based on the combination of SERDUX and MARCIM. The SERDUX method includes a Susceptible node (S), an Expected node (E), a Recovery node (R), a Degraded node (D), an Inaccessible node (U), and a destroyed node (X). MARCIM is also related to the maritime cyber defense framework. This method is evaluated from two perspectives: 1) sustainability and performance, 2) real-world scenario. The evaluation results show that this model has been able to improve the awareness and development of solutions to deal with threats.

L. Q. Sánchez *et al.* [11] present an SEIRS-NIMFA method based on a delay period for malware propagation. In this method, a Markov chain approach, called n-interlocked mean-field approximation, is used to reduce the complexity, which is sensitive to the network structure. This method is individual-based, which distinguishes the individual state of each device from the other by examining the overall evolution of the network, which makes it not based on population density.

G. Wu *et al*. [12] presented a worm propagation method called SIHQR using time delay and expressed the level of infection through a game model between sensitive and infected nodes. The SIHQR method includes 5 nodes. This paper examines the conditions of disease-free equilibrium, endemic equilibrium, and Hopf bifurcation.

A. M. Rey [13] presented a segmented and global spread model of malware. This model includes S (susceptible), S_P_ (patched susceptible), L (Latent non-infectious), L_I_ (Latent infectious), A (Compromised non-infectious), A_I_ (Compromised infectious), D (Damaged), Q (Deactivated) nodes. This model can determine the duration of the outbreak based on the definition of the unit of time by different nodes, and the reproduction number is explicitly calculated.

E. Asadi and S. Hosseini [14] presented a malware propagation and clustering model. This method includes nodes S (Susceptible), E (Exposed), I (Infected), and R (Recovered). In this model, the basic reproduction rate (R0) is calculated to obtain the consequences of malware propagation, and clustering is also used to reduce the spread of malware. The evaluation results show that this method has been able to reduce the infection rate.

A. Chernikova *et al.* [15] proposed a propagation model for self-propagating malware (SPM). The model consists of nodes S (Susceptible), I (Infected), I_D_ (Infected Dormant), and R (Recovered). This model investigates how to extract the reproduction number and disease-free equilibrium points. This model extracts the transmission rates of 15 attacks in WannaCry, and the evaluation results show that it performs better on real data.

Comparative epidemiological models have been used in the past to model computer malware in scale-free networks. In these models, each node is assumed to be in one of the susceptible, infected, or recovered states, and the transmission rate is constant in time, and temporary immunity is ignored. The SEIRVS model overcomes these limitations by adding two states, Vaccinated and Susceptible-again. This model is able to simulate real-life behavior of threats by re-sensitizing due to reduced immunity and taking into account the effect of vaccination.

### 2.2. Intrusion Detection System (IDS)

In this section, intrusion detection systems, combined methods of metaheuristic algorithms and machine learning proposed in the past few years are described.

A. Al. Mazroa *et al.* [16] presented a hybrid method based on deep learning algorithms. In this method, first, Z-score normalization is used for preprocessing, second, feature selection is performed using Binary Gray Wolf Optimization (BGWO). Third, the Element Enhanced Spike Neural Network (EESNN) is used to detect attacks, and fourth, the Archimedes Optimization Algorithm (AOA) is used for hyperparameter selection. This method has been evaluated using the NSLKDD2015 and CICIDS2017 datasets. The evaluation results show that the proposed method has achieved an accuracy of 99.12% and 99.36%.

E. I. Elsedimy and S. M. M. AboHashish [17] presented a hybrid method based on fuzzy and metaheuristic algorithms called FCM-SWA. In this method, first, the performance of the Fuzzy C-means method (FCM) is improved by using the Sperm Whale algorithm (SWA). Second, the adaptive threshold method is used to increase the global search performance of SWA. The proposed method is evaluated using the NSL-KDD, AWID, and BoT-IoT datasets. The evaluation results show that the proposed method can overcome the clustering problems.

M. Antonijevic *et al*. [18] presented a hybrid method based on deep learning algorithms. In this hybrid method, firstly, it combines the algorithms of Convolutional Neural Networks (CNN), Classification Boosting (CatBoost), and Light Gradient Boosting Machine (LightGBM). Then, the basic chimp optimization algorithm (ChOA) method is used to tune the parameters. The proposed method is evaluated using a realistic data set. The evaluation results show that the proposed method detects attacks with an accuracy of 0.998.

E. Akhmetshin *et al.* [19] presented a detection method based on metaheuristic optimization algorithms for detecting Denial-of-Wallet (DoW) attacks. In this method, first, the dataset is normalized using the z-score method. Second, the Harris Hawke Optimization (HHO) algorithm is used for feature selection. Third, a combination of Gated Recurrent Unit (GRU), Temporal Convolutional Network (TCN), and Convolutional Autoencoder (CAE) algorithms is used to detect attacks. Fourth, the Modified Marine Predator Algorithm (MMPA) is used to tune the cloud parameters. The evaluation results show that this method detects attacks with an accuracy of 0.981.

T. Vaiyapuri *et al.* [20] presented a hybrid method based on metaheuristic algorithms. In this method, first, the min-max scalar is used for data normalization; second, the combination of the Harris Hawks algorithm with the cosine algorithm is used for the feature selection process. This method is used to detect attacks. Fourth, the Gazelle Optimization Algorithm (GOA) is used to improve the results. The evaluation results show that the proposed method performs better than other deep learning methods.

S. Thota and D. Menaka [21] presented a detection method using deep learning algorithms. This method is based on the combination of a convolutional neural network and pelican optimization system. The evaluation results show that this method can evaluate attacks with an accuracy of 0.995.

H. R. Sayegh *et al.* [22] presented a hybrid method based on deep learning and metaheuristic algorithms. In this method, flower pollination algorithms (FPA) and deep neural networks (DNN) are combined to detect attacks. In this method, the Bagging algorithm is used to solve the problem of unbalanced class distribution, and the DNN algorithm is used for base learners. The proposed method is evaluated using NSL-KDD, UNSW NB-15, CIC-IDS 2017, and BoT-IoT datasets.

A. S. Mashaleh *et al.* [23] present a hybrid approach based on fuzzy logic and metaheuristic algorithms. In this approach, uncertainties and ambiguities are addressed using fuzzy logic, and the parameters of the fuzzy method are tuned using the particle swarm optimization (PSO) algorithm. The proposed approach is evaluated using the CICIoT2023 dataset, and the evaluation results show that this approach is suitable for high-severity threats.

HM. Fadhil *et al.* [24] presented a hybrid method based on metaheuristic algorithms. In this method, it is based on the Lion Optimization Algorithm (LOA) and the Gray Wolf Optimizer (GWO). In this method, first, the Lion Optimization Algorithm (LOA) is used for feature selection and the GWO algorithm is used to reduce the parameters of an infiltration mechanism. The proposed method is evaluated using the NSL-KDD dataset.

R. Ghanbarzadeh [25] presented a hybrid method for attack detection. In this method, the Horse Herd Optimization (HOA) algorithm is used for feature selection. In the HOA algorithm, it is converted into a discrete algorithm using the floor function and then optimized using the concepts of quantum computing. Finally, the K-Nearest Neighbor (KNN) algorithm is used to detect attacks. The evaluation results are evaluated using the NSL-KDD and CSE-CIC-IDS2018 datasets. The evaluation results show that the proposed method has achieved an accuracy of 99.8%.

M. Otair [26] presented a hybrid method of machine learning and metaheuristic algorithms. In this method, the Gray Wolf Optimization (GWO) algorithm is used for feature selection, the Particle Swarm Optimization (PSO) algorithm is used for updating information, and the k-means algorithm is used to detect attacks on the NSL KDD dataset to verify the performance of the proposed technique. Classification is performed using the k-means and SVM algorithms to measure the performance in terms of accuracy, detection rate, false alarm rate, number of features, and execution time. The results have shown that the proposed technique has achieved the necessary improvement over the GWO algorithm when using the K-means or SVM algorithms.

X. Zhang and Z. Wei [27] presented a hybrid method based on metaheuristic algorithms and machine learning for solar radiation detection. In this method, the dimensions of the data set are first reduced using the principal component analysis (PCA) algorithm, and then training is performed using the bat algorithm. Based on four series of solar radiation, the experimental simulation shows the effectiveness of the model.

W. Guo *et al.* [28] presented an improved Whale Optimization Algorithm. The Whale Algorithm has a slow convergence rate and gets stuck in a local optimum solution. In order to overcome this problem, it uses a binary method using adaptive neighborhood strategies and hybrid mutation. This method has been evaluated using twelve standard datasets from the UCI repository. The evaluation results show that this method improves the exploitation ability, population quality, and convergence speed.

W. Fu *et al.* [29] presented a hybrid method for detecting rolling bearing faults. In this method, first, the variable mode decomposition (VMD) approach was used to analyze the signals, and the combination of the sine cosine algorithm (SCA) and the periodic jump method was used to improve the performance of the Harris Hawkes optimization (HHO) algorithm, and finally, the support vector machine (SVM) algorithm was used for classification. The performance of this method was evaluated using four reliability indices. The evaluation results show that this method has achieved satisfactory detection results.

H. Bayati *et al.* [30] presented a feature selection method based on the particle swarm algorithm. In this method, a new filter method is based on the particle swarm optimizer (PSO) in which the initial population is first generated and divided into two equal groups, and the pairs compete and the winners move on to the next stage, and in each iteration, the objective function is calculated for all particles, and the best subset of features is selected. The evaluation results show that the proposed method has performed better than other methods.

A. Ibrahim and M. Tawhid [31] presented a hybrid method for Nebdi classification. In this method, first feature selection is performed using the binary version of the Bat Algorithm (BA) and then the Differential Evolution (DE) algorithm is used to explore the feature space. The proposed method is evaluated using a dataset obtained from the UCI repository. The results show that the proposed algorithm has high ability.

S.Dutta *et al.*[32] A hybrid method based on the Whale and Bat optimization algorithms has been presented. In this method, the Whale optimization algorithm is first modified and then the Bat algorithm is used to increase accuracy and training. The proposed method is simulated using the Modelsim method.

M. Sofiane and D Zouache [33] presented the firefly algorithm for feature selection. In this method, to improve the firefly algorithm, a new formula was used to calculate the attractive distance r and A. Experimental results show that the proposed method performed better than other methods.

A. Kaur et al. [34] presented a hybrid method for detecting attacks. In this method, a new hybrid method for anomaly detection based on K-Means and firefly algorithms has been presented. In this method, clustering has been used for training. The proposed method has been evaluated using the NSL-KDD dataset. The evaluation results show that this method has performed better than other methods by a large margin.

X. Zhao *et al.* [35] presented a binary method of the differential evolution algorithm for feature selection. In this method, a two-stage approach is used, which is based on three methods of Fisher’s score, T-statistic, and information gain for feature selection, which are given as input to the differential evolution (DE) algorithm. In this method, the support vector machine (SVM) algorithm is used for classification. Experimental results show that this method has high potential.

E. Emary *et al.* [36] presented a binary method for gray wolf. The method is based on two steps. In the first step, individual steps are taken towards the three best solutions, and the binary position of the gray wolf is updated using the random intersection between the three moves. In the second step, a sigmoid function is used to update the position. The method has been evaluated using 18 datasets from the UCI repository.

These methods are overly specific and computationally expensive in real-world, large-scale environments. The proposed IDS uses the SEIRVS model to provide a predictive approach to threats. The proposed model performs rapid parameter optimization without heavy training, which reduces computational cost and increases accuracy. These methods often have high convergence times and randomly select initial conditions, which may not achieve global convergence. The Golden Ratio Optimization (GRO) algorithm uses the concept of the golden ratio in the search space to quickly optimize the optimization path and reduce the number of objective function evaluations, speed limits, and instability.

## 3. Proposed model

In this paper, a method of epidemic of botnet attacks like path diseases called SEIRVS (susceptible-Exposed-Infected-Recovery-Vaccinated) in a heterogeneous environment is presented. Here, an intrusion detection system is also presented to identify botnet attacks using a combination of machine learning and metaheuristic algorithms. This article presents two distinct but complementary roles: an epidemic model and an intrusion detection system. The first role involves an epidemic model in which the macro behavior, spread rate, and critical points of attack propagation dynamics at the network level are examined. The second role involves an independently designed intrusion detection system (IDS) that does not directly use the epidemic model in the practical implementation of the detection process. However, the results and insights derived from the epidemic model are used to provide an analytical foundation to guide the interpretation of the IDS’s performance. Therefore, although both roles are independent in terms of implementation, they are conceptually related and complement each other in enhancing understanding and countering attacks. In the proposed IDS, first, the data set is preprocessed; secondly, using the Golden Ratio (GRO) algorithm, the feature set with the most fitness value is selected as the best features; and thirdly, using the Bat Algorithm (BA) and K-Nearest Neighbor (KNN) algorithms, the attacks are identified, and this method is called GRO-BA-K-NN.

### 3.1. Proposed epidemic model

**Fig 2:**
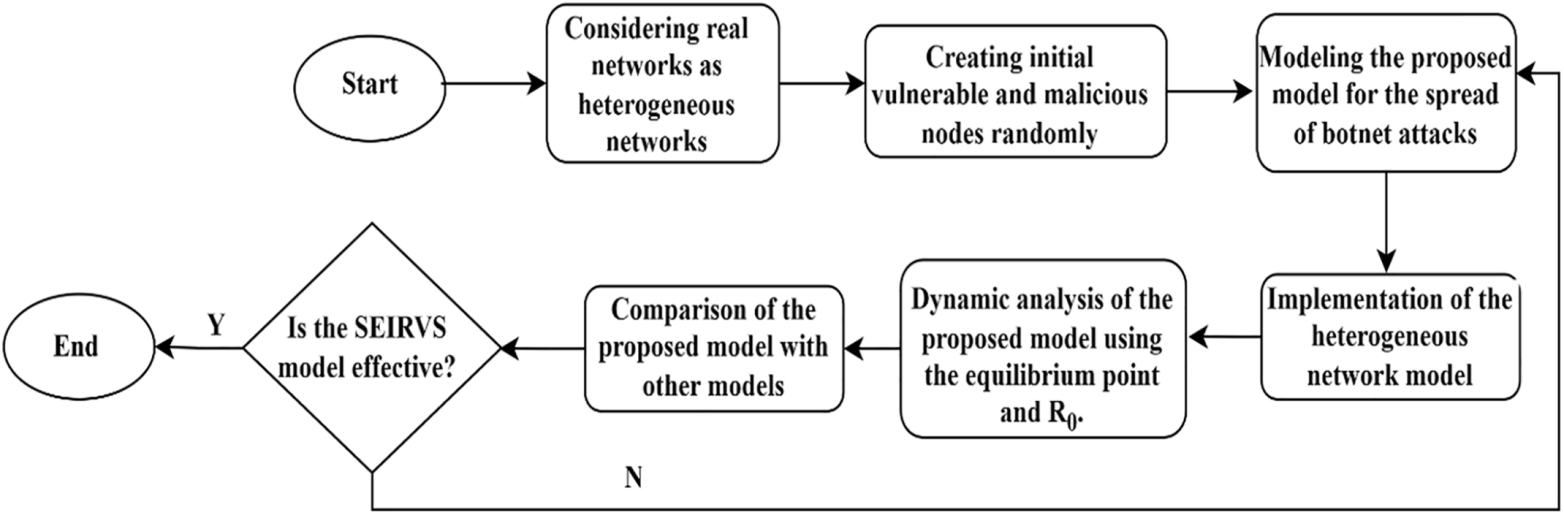
Diagram of the modeling and evaluation steps of the SEIRVS model in heterogeneous networks.

Fig 2 shows the overall flow of the modeling and evaluation steps of the proposed SEIRVS model for analyzing the spread of botnet attacks in heterogeneous networks. First, real networks are considered heterogeneous networks, and then initial vulnerable and malicious nodes are created. Finally, the equilibrium point and the baseline value are used to compare and demonstrate the superiority of this method.

**Fig 3:**
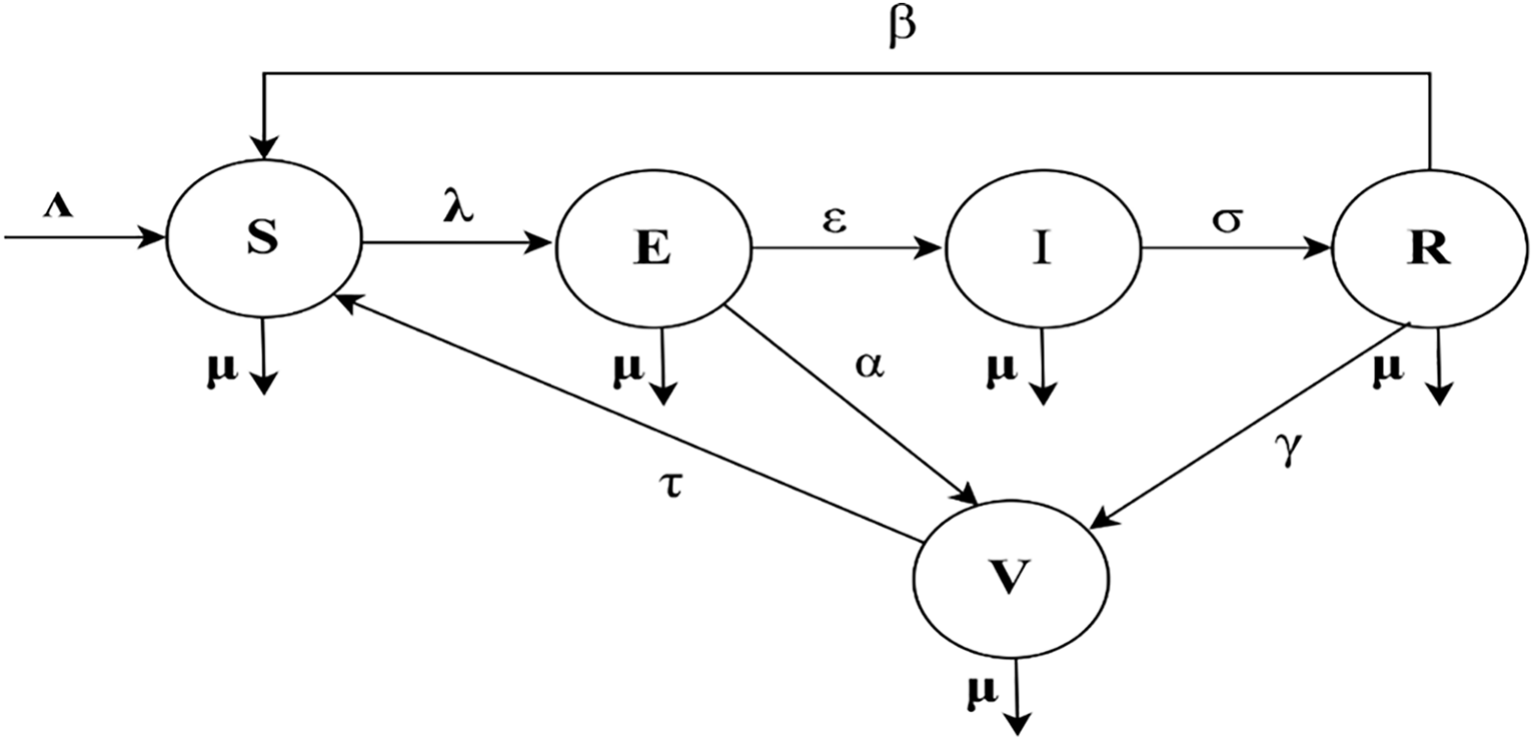
State transition of the SEIRVS model.

This paper presents the SEIRVS model, which works for botnet attacks by simulating how malware spreads, similar to modeling epidemics in a population. Fig 3 shows the state transition diagram of the SEIRVS model.

#### 3.1.1. Model description.

This model divides network nodes into five groups: susceptible (S), exposed (E), infected (I), recovered (R), and vaccinated (V).

S: Includes devices vulnerable to infection that can become part of a botnet. All new devices that are connected to the network but have not yet been infected are in this group.

S → E: When a vulnerable device communicates with an infected device and receives the infection, the device moves from the vulnerable state to the vulnerable state.

E: Includes devices that have been contaminated but do not exhibit abnormal behavior.

E → I: The device’s dormant phase has ended, and the device begins to behave maliciously through user interaction, making it contagious.

I: Infected devices that are active in spreading the infection to other devices.

I → R: When security measures (such as antivirus or network scanning) are applied to devices, the infected device is cleaned, and further infection is prevented.

R: Includes devices that are now protected and are resistant to re-infection for a period of time and are no longer considered a source of infection.

R → V: At this stage, the recovered devices are vaccinated and cleaned using more advanced security measures (such as intrusion detection systems).

V: It includes devices that are not only clean but also vaccinated with multiple layers of security and have the ability to detect and prevent new infections.

R → S: Devices can lose their immunity and become vulnerable again due to reasons such as delayed updates, infection development, and antivirus expiration.

E → V: In some cases, hidden infected devices can be prevented from spreading their infection by using specific security measures and moved directly to the vaccinated stage.

V → S: Over time or due to the development of new threats, vaccinated devices lose their strength and become vulnerable devices.

In the proposed model, two types of node dynamics are considered: node removal with an exit rate μ and node addition with a connection rate ʌ, which are based on the density of nodes of degree k. To maintain the structural stability of the network, the addition and removal of links through leaf connections is balanced, and all nodes remain stable over time. So, the number of connections is equal to the number of leaves, and both operations occupy a single edge in the network structure. Table 2 presents the symbols used in the SEIRVS model.

**Table 2:** Symbols of the model.

Notation	Description
$S_{k}$(t)	Number of susceptible nodes of degree k at time t in the network
$E_{k}$(t)	Number of vulnerable nodes of degree k at time t in the network
$I_{k}(\text{t})$	Number of infected nodes of degree k at time t in the network
$R_{k}$(t)	Number of recovered nodes of degree k at time t in the network
$V_{k}$(t)	Number of vaccinated nodes of degree k at time t in the network
Λ	The rate at which new nodes are added to the network, these new nodes become susceptible. (Λ > 0)
μ	The rate of nodes leaving the network, which has the same proportion in all cases, in other words, includes the death rate. (μ>0)
*λ*	The rate of infection of susceptible individuals per person per unit of time with contact with infected individuals, based on the contact rate and probability of transmission (network spread rate) 0 < **λ ≤** 1
ε	The transfer rate from the exposed node to the infected node. 0 < ε **≤** 1
σ	The rate at which infected individuals recover and move into the recovered category at any given time. 0 < σ **≤** 1
γ	The rate at which recovered individuals are vaccinated and transmitted to vaccinated nodes. 0 < γ **≤** 1
α	The rate at which individuals in the exposed group are vaccinated and transmitted to the vaccinated node. 0 < α **≤** 1
τ	The transfer rate from the vaccinated node to the susceptible node. 0 < τ **≤** 1
β	The transfer rate from the recovery node to the susceptible node. 0 < β **≤** 1

During the process of botnet attacks spreading in the network, these nodes change their state using the following rules:

1) All newly connected devices are immune to infection. Devices connect to the network at a constant positive rate ʌ and disconnect from the Internet at a rate μ, and newly connected devices are assumed to be immune to infection.
2) A node in the susceptible state is attacked by infected devices, but is not immediately infected and can become an infected node at a rate λ.
3) Once infected, devices are placed in two states: latent or spreading. Vulnerable nodes follow two possible paths: if they behave suspiciously, they are vaccinated at a rate α to protect themselves from infection and prevent further spread. If they are hidden, they are infected at a rate ε and transition to state I.
4) At node I, infected devices recover partially through antivirus mechanisms at a rate σ and move to state R.
5) Security measures such as antivirus software are designed to protect against a specific type of botnet attack, since this immunity is temporary due to the evolution of botnets over time. Recovered devices can be vaccinated at a rate of 𝛾, and by incorporating security measures such as an intrusion detection system (IDS), the speed and number of infected nodes can be reduced and their protection extended.
6) If the antivirus protection expires before vaccination, the nodes can become susceptible again at a rate 𝛽. Similarly, due to the evolution of attacks, there is no permanent immunity. Therefore, recovered devices can have a temporary immunity period and become susceptible again at a rate τ.

Assumptions in modeling are a critical issue for simplification and problem solving. The following are the assumptions of the proposed model. In modeling, it is necessary to eliminate variables that have the least impact on the system, which can be of great importance in simplifying the equations. The assumptions of the proposed model are given below. The following can be considered in the proposed epidemic model:

1. It is assumed that many diverse and anonymous botnet attacks are generated.
2. The network topology is based on a Barabasi-Albert (BA) neural network, which is a heterogeneous network.
3. In this experiment, the number of nodes is assumed to be 1000.
4. The number of nodes is fixed over time; that is, the birth rate is balanced with death. The links are balanced by leaves, so ᴧ = *μ*, The parameter $N_{k}(t)$ is defined as the ratio of the total number of nodes with degree *k* at time *t* over all states of susceptible, exposed, infected, recovered, vaccinated. Due to the uneven degree distribution in networks, all network members are divided into k groups based on the degree of the network members. The network members in the kth group have the same degree distribution and number of connections as the others. It is assumed that $S_{k}$(t), $E_{k}$(t), $I_{k}$(t), $R_{k}$(t) and $V_{k}$(t) are the densities of network members with degree k in the states V, S, E, I, R for k = 1, 2,..., n. The sum of these ratios in each degree class is equal to 1, in other words: $N_{k}(t)=\;S_{k}(t)+E_{k}(t)+\;I_{k}(t)+R_{k}(t)+V_{k}(t)\equiv \text{1}.$
5. The death rate *μ* is considered the same and constant for all nodes.
6. At the start, 100 nodes are infected, which act as seeds, and the rest of the nodes are susceptible.
7. How the infected nodes are initially selected determines the malware propagation method. Here, infected nodes are randomly selected for the propagation process.
8. We consider dangerous botnet attacks and consider any security measure to be ineffective over time due to the spread of attacks.
9. The recovery phase can provide a suitable time for vaccination, but sometimes new attacks prevent it from being vaccinated, and it becomes a susceptible node again.
10. To increase the accuracy of the simulations, all simulations are performed in 20 iterations.

#### 3.1.2. Model formulation.

Here, the SEIRVS analytical model is introduced to investigate the dynamics of botnet attack propagation in heterogeneous networks based on differential equations. At the beginning of the botnet attack propagation process, there are 100 infected nodes, and the rest are sensitive nodes. The initial state of botnet attack propagation is described below.


$$\begin{array}{c} S_{k}(\text{0})\;\approx \text{ 900},E_{k}(\text{0})\;\approx \;\text{0},\;I_{k}(\text{0})\;\approx \text{ 100},\;R_{k}(\text{0})\;\approx \;\text{0},\;V_{k}(\text{0})\;\approx \;\text{0}. \end{array}$$

The following is the formula for the spread of attacks in heterogeneous networks:

When a botnet attack occurs in a network, the number of nodes that were previously susceptible and are now latently infected due to contact with the infection and are at risk decreases with the attack propagation rate. Based on the rates of connection and leaving the susceptible state, it is calculated using equation (1).


$$\begin{array}{c} \frac{dS_{k}(t)}{dt}=\Lambda +\beta R_{k}(t)+\tau V_{k}(t)-\lambda S_{k}(t)\theta -\mu S_{k}(t) \end{array}$$ (1)

In equation (1), the θ, parameter indicates how contamination spreads in a network and is calculated using equation (2).


$$\begin{array}{c} \theta =\frac{\text{1}}{\left\langle k\right\rangle }\sum_{k=m}^{z}kP(k)I_{k}(t) \end{array}$$ (2)

Equation (2) formulates how contamination spreads in a network where nodes have different degrees. In this equation, the parameter *k* is the degree of the node, which is the number of direct connections of that node in the network, whose values vary between m (minimum degree) and *z* (maximum degree). The parameter P(k) is the degree distribution function, which indicates the probability that a node is randomly selected. $I_{k}$ is the density of infected nodes of degree k at time 𝑡, and (k) is the average number of connections per node, calculated using Equation (3).


$$\begin{array}{c} \left\langle k\right\rangle =\sum_{k=m}^{z}kP(k) \end{array}$$ (3)

In equation (3), the degree of each member of the Internet network is considered as m = 1 ≤ k ≤ n, where m is the lowest degree, and n is the highest degree. P(k) is the degree distribution of all members of the Internet network. And as a result, $\sum_{k=m}^{n}P(k)=\text{1},$ According to equation (1), the vulnerable nodes are attacked by infected nodes, leaving the infected node and adding it to the vulnerable nodes. The vulnerable nodes, after the end of the incubation period, show infection and infect neighboring sensitive devices at a rate ε. Nodes at risk are vaccinated at a rate of α if they exhibit suspicious behaviors. This is calculated using equation (4).


$$\begin{array}{c} \frac{dE_{k}(t)}{dt}=\lambda S_{k}(t)\theta -\epsilon E_{k}(\text{t})-\alpha E_{k}(\text{t})-\mu E_{k}(t) \end{array}$$ (4)

When a node is infected, infected nodes are removed from the vulnerable node at a rate of ɛ and added to the infected nodes at time t. And when an infected node is recovered, it uses temporary security methods to provide time for vaccination. Infected nodes are removed from the infected nodes at a rate of σ and added to the recovered nodes. This is done using equation (5).


$$\begin{array}{c} \frac{dI_{k}(t)}{dt}=\epsilon E_{k}(\text{t})-\sigma I_{k}(\text{t})-\mu I_{k}(t) \end{array}$$ (5)

When nodes are infected, infected nodes are recovered at a rate of σ using temporary methods to provide time for vaccination. During recovery, if for reasons such as the spread of a stronger attack or the expiration of the antivirus time, it is possible for the detected node to be transferred to a sensitive node at a rate of β, otherwise, the node is vaccinated at a rate of γ using intrusion detection system methods. This is done using equation (6).


$$\begin{array}{c} \frac{dR_{k}(t)}{dt}=\sigma I_{k}(\text{t})-\gamma R_{k}(\text{t})-\beta R_{k}(t)-\mu R_{k}(t) \end{array}$$ (6)

Of course, due to the spread of attacks, no security method can be permanent, so over time, the vaccinated nodes at a rate of τ become sensitive nodes again. This is done using the equation (7).


$$\begin{array}{c} \frac{dV_{k}(t)}{dt}=\gamma R_{k}(\text{t})+\alpha E_{k}(\text{t})-\tau V_{k}(t)-\mu V_{k}(t) \end{array}$$ (7)

### 3.2. Proposed intrusion detection system

**Fig 4:**
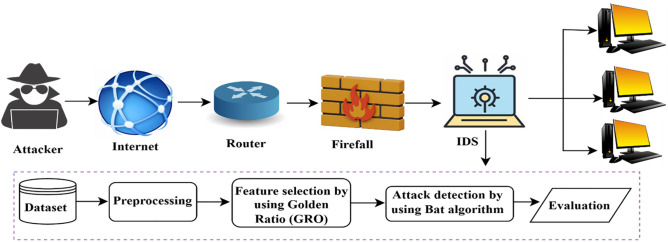
Scheme of the GRO-BA-K-NN method.

This paper presents a hybrid IDS based on metaheuristics, machine learning algorithms, which is based on 3 algorithms: the Golden Ratio Optimization (GRO) algorithm, Bat Algorithm (BA) and K-Nearest Neighbor (KNN) algorithms. This method consists of 3 steps: 1) preprocessing, 2) feature selection, and 3) attack detection. The general framework of the GRO-BA-K-NN method is shown in Fig 4. Fig 4 shows a process flow of cyber attack detection based on optimization and machine learning algorithms. It is divided into two parts; the upper part shows from the start of the attack to detection. In the beginning, the hacker starts the cyber attack, and the data and traffic are transferred in the network, which may be infected or healthy. Next, the router acts as a network router, where the data is directed, and firewalls are protective walls to prevent unauthorized traffic from entering. Firewalls may not be able to prevent unauthorized entry, in which case the attack detection system detects and analyzes the attacks. These systems prevent them from entering the target systems by giving timely warnings. In the lower part, the steps of the proposed attack detection system are given, in which first the data set in question is preprocessed, then feature selection is done using the Golden Ratio Optimization (GRO) algorithm, then training is done using the Bat algorithm, and finally identification is done using the K-NN algorithm.

#### 3.2.1. Preprocessing.

In preprocessing, raw data is prepared for model analysis, which consists of 3 steps. This step includes 1) Data cleaning: replacing missing data using the mean of numerical data, identifying outliers using the Z-score method, and removing them. 2) Converting non-numeric data to numerical data. Here, using the Min-Max method, all features are transformed in the range [0, 1] using equation (8).


$$\begin{array}{c} \text{Z}=\frac{a-\text{min}(\text{a})}{\text{max}(\text{a})-\text{min}(\text{a})} \end{array}$$ (8)

By preparing the dataset, it is divided into two parts: training and testing. Here, 70% is considered for training, and the other 30% is considered for testing.

#### 3.2.2. Feature selection.

All elements in nature have a similar pattern in different shapes and sizes, and each of them follows a constant physical ratio called the Golden Ratio Optimization (GRO). In IDS, the dataset contains hundreds of features, but not all of these features are needed. The number of features increases the computational cost. The goal of using the GRO algorithm is to find a suitable subset of features that increases the detection accuracy and reduces the computational cost. In GRO, instead of searching randomly, the search space is scanned numerically between [0,1] based on the golden ratio, and each position is a subset of features. This ratio was introduced by Fibonacci. This ratio consists of a series of numbers that are the sum of the two numbers before them. Equation (9) describes this property.


$$\begin{array}{c} P(n)=GF*\frac{\varphi^{n}-(\text{1}-\varphi {)}^{n}}{\sqrt{\text{5}}}\;where\;GF=\text{1}.\text{6}\text{1}\text{8} \end{array}$$ (9)

In equation (9), the parameter φ is the ratio between two consecutive numbers. Here, each solution is a vector that is first updated by the population mean and then the fit value to determine the worst and best solution. To increase the convergence speed, the mean value is updated. Second, a random population is generated, and the effect of the movement on the best and worst vectors is shown to move one step forward towards the optimization. To detect the overall position of the population, the population mean is calculated. This substitution is done using equations (10) and (11).


$$\begin{array}{c} F_{best}>F_{medium}>F_{worst} \end{array}$$ (10)

$$\begin{array}{c} {\vec{X}}_{t}={\vec{X}}_{medium}-{\vec{X}}_{worst} \end{array}$$ (11)

Each solution vector 𝑋 = [$X_{\text{1}},X_{\text{2}},\ldots ,X_{n}$] is a subset of features in the dataset. In equation 20, parameter X represents the position of a solution in the search space. In the proposed IDS, each 𝑋 is a subset of selected features, and parameter F in equation 10 represents the fitness value of each 𝑋. This value evaluates the quality of the selected feature subset. To create variety in order to reach the global solution and avoid local optima, a random move is added. This move is calculated using equation (12).


$$\begin{array}{c} {X\;}_{new}=\left(\text{1}-F_{t}\right)X_{best}+rand*F_{t}*X_{t} \end{array}$$ (12)

The rand parameter is a random number drawn from a uniform distribution over the time interval [0,1], i.e., r and ∼U(0,1), and $F_{t}\;$ is a control factor that adjusts the influence of the attack-induced disturbance on the state update. In random walk, if $F_{t}$ is small, the coefficient $F_{t}-\text{1}$ becomes large and the influence of $X_{best}$ on ${X\;}_{new}$ increases, meaning the walk moves towards the best solution. If F is large, the $rand*F_{t}*X_{t}\;$part becomes more important and the random variation increases with respect to the current solution. The rand part causes a different value of the input vector $X_{t}$ to be combined each time the equation is executed, thus preventing getting stuck in a local optimum.

Now, by examining the boundary conditions, a new solution is substituted.


$$\begin{array}{c} X^{i}=\overset{i}{\underset{new}{X}}\;if\;\overset{i}{\underset{testpoint_{new}}{F}}<\overset{i}{\underset{testpoint}{F}} \end{array}$$

$$\begin{array}{c} X^{i}=\overset{i}{\underset{old}{X}}\;otherwise \end{array}$$ (13)

Now, the golden ratio is used to approximate the best answer, which is calculated using equation (14).


$$\begin{array}{c} X_{new}=X_{old}+rand*\left(\frac{\text{1}}{F}\right)*\left(X_{best}-X_{worst}\right)\;\left(\frac{\text{1}}{F}\right)=\text{0}.\text{6}\text{1}\text{8} \end{array}$$ (14)

**Fig 5:**
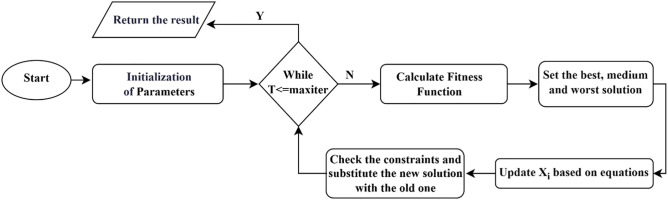
The flowchart for the GRO algorithm.

Fig 5 shows the proposed algorithm for feature selection, and Algorithm 1 shows the code of the proposed method.

**Algorithm 1: GRO**

Input: Data set

Output: Best features

1. Preprocessing the dataset

2. Initialize population

3. While T<=maxiter

4.  Calculation fitness

5.  Based on the fitness function X_meduim_, the average value of all solutions and the worst fit X_worst_ are obtained.

6.  if Fit (X_meduim_) <Fit (X_worst_) then

7.  X_meduim_ = X_worst_

8.  Generate random population

9.  Compare all populations and update X_best_

10.  Check the constraints and substitute the new solution with the old one

11. Return best features

#### 3.2.3. Attack detection.

**Fig 6:**
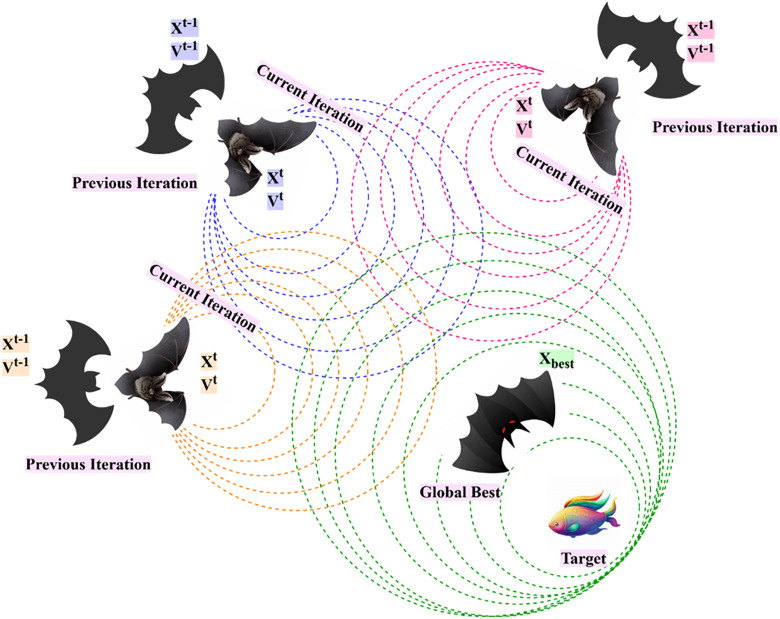
Bat algorithm performance.

The main feature of bats is their eyesight, which has a high ability for echolocation. The Bat Algorithm (BA) is a metaheuristic algorithm that simulates bat echolocation, which was presented by Xin Shiyang [37] in 2010. Bats use the reflection of a long and short sound pulse to their ears, and the time difference between the transmission and reflection of these signals helps bats determine their distance to an object. Using this method, bats can distinguish between an obstacle and prey, and can even hunt in complete darkness. The algorithm works according to the following rules: 1) Bats can sense the distance and distinguish between prey and obstacles using echolocation. 2) Bats move randomly at a speed v at a position x. 3) They send signals with different wavelengths and heights of a fixed frequency range. Based on their proximity to the target, they determine the pulse propagation rate r ∈ [0.1] and vary the wavelength of the pulses. 4) The loudness can be changed using several methods; the loudness is reduced from maximum to minimum. The performance of the bat algorithm is shown in Fig 6.

Initially, the velocity and frequency are initialized. At each iteration for the i-th bat, their velocity and position are calculated using equations (15) and (16).


$$\begin{array}{c} \overset{t}{\underset{i}{V}}=\overset{t-\text{1}}{\underset{i}{V}}+(\overset{t}{\underset{i}{X}}-X^{*})f_{i} \end{array}$$ (15)

$$\begin{array}{c} \overset{t}{\underset{i}{X}}=\overset{t-\text{1}}{\underset{i}{X}}+\overset{t}{\underset{i}{V}} \end{array}$$ (16)

In equation (15), the expression x* represents the global bat (best solution) at the current moment. The speed update is done by multiplying $f_{i}$, which is calculated using equation (17).


$$\begin{array}{c} f_{i}=f_{min}+(f_{max}-f_{min})\beta \end{array}$$ (17)

In equation (17), the parameter β is a random vector in the interval [0,1] that can set the value of the parameter $f_{i}$ randomly between $f_{min}$ and $f_{max}$. This variation causes the algorithm to obtain different values of f in each iteration or for each member of the population and helps in exploring the search space. Local search is a random search around the current optimal solutions, calculated using equation (18).


$$\begin{array}{c} X_{new}=X_{old}+\epsilon A^{t} \end{array}$$ (18)

In equation (18), where the parameter $X_{new}$ represents the updated position of the solution (a bat) in the current iteration, the parameter $X_{old}$ represents its position in the previous iteration. The parameter ε is a random number drawn from a uniform distribution on the interval ([0,1]) that ensures moderate step sizes and also reduces the risk of premature convergence to local optima. The expression $A^{t}\;=<\overset{t}{\underset{i}{A}}>$ represents the average loudness of the bat population at iteration (t). In this algorithm, to represent the balance between exploration and exploitation, the loudness of the bats decreases and the pulse propagation rate increases as they approach the prey. The update rules for loudness and pulse propagation rate are defined in equation (19).


$$\begin{array}{c} \overset{t+\text{1}}{\underset{i}{r}}=\overset{\text{0}}{\underset{i}{r}}\left[\text{1}-\text{exp}(-\gamma t)\right]*\overset{t+\text{1}}{\underset{j}{A}}=\beta \overset{t}{\underset{i}{A}} \end{array}$$ (19)

In equation (19), the values of the parameters β and γ are constant, with the parameter γ being greater than 0 and the parameter β between 0 and 1.


$$\begin{array}{c} \overset{t}{\underset{i}{A}}\to \text{0},\;\overset{t}{\underset{i}{r}}\to \overset{\text{0}}{\underset{i}{r}}\text{ as }t\to \infty \end{array}$$

The K-NN algorithm is used to detect attacks.

**Fig 7:**
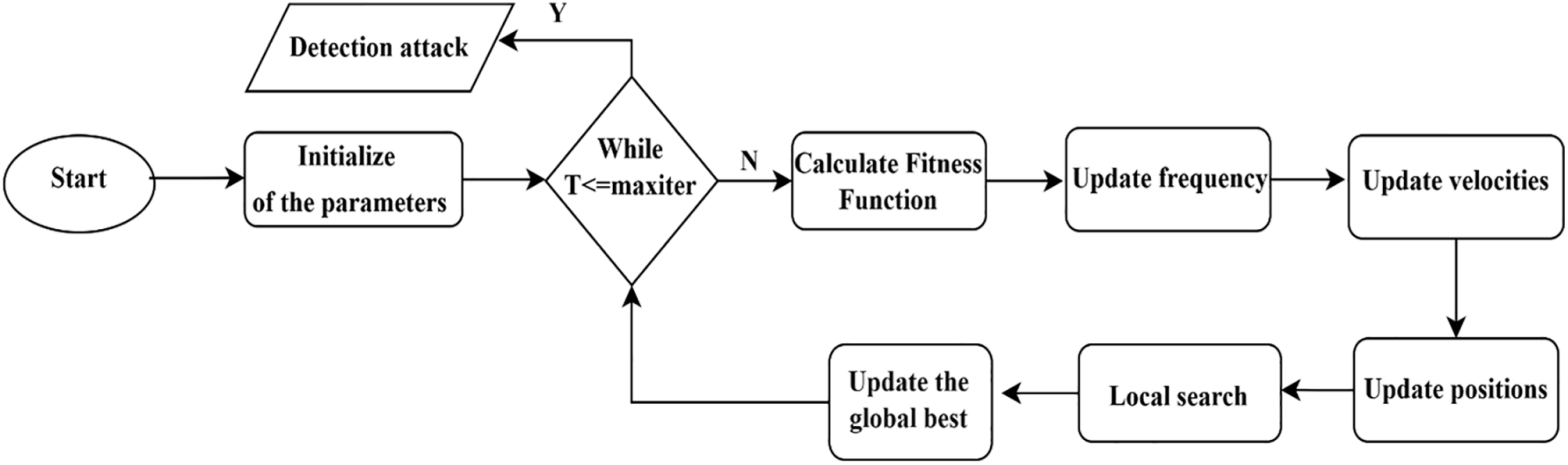
The flowchart for the BA algorithm.

Fig 7 shows the proposed algorithm for attack detection, and Algorithm 2 shows the code of the proposed method.

**Algorithm 2: BA**

Input:Subset features

Output: Classiffication

1. Initialize of the parameters

2. While T<=maxiter

3.  For I = 1….N do

4.   Update frequency

5.   Update velocities

6.   Update positions

7.  If rand > r_i_ then

8.   Select best solution

9.   Local search

10.  End

11.  If rand < A_i_ and f(X_i_) < f(X_best_) then

12.   Accept new solution

13.  End

14. Classification attacks

Table 3 describes the parameters used by GRO-BA.

**Table 3:** Setting the parameters of the proposed method.

Parameter	Value	Parameter	Value
cross	5	cross	5
popSize	20	popSize	10
maxIter	100	maxIter	100
A	0.5	omega	1
r	0.5		
frequency_min	0		
frequency_max	2		
w_min	−10		
w_max	10		
b_min,	−10		
b_max	10		

The bat algorithm is based on an optimization algorithm where each bat moves through the search space to find the best solution and each solution contains the parameters of the KNN machine learning model. In other words, the bat algorithm is used to adjust the parameters of the KNN model. In the bat algorithm, the speed and position of the bats are changed to reach the most optimal solution in the search space. Here, position (X) represents the values of the KNN parameters, and velocity (v) represents the rate of change in the parameter settings per iteration. The Bat-K-NN hybrid method is an optimized version of the K-NN algorithm that uses the Bat algorithm to optimize the parameters of the K-NN algorithm and overcome the weaknesses of the classic K-NN algorithm. The reasons for choosing the Bat-K-NN method over other methods are: 1) Improving the performance of the K-NN algorithm because the simple K-NN is very sensitive to the value of 𝐾, which the Bat algorithm optimizes here, thereby increasing the accuracy and reducing the error. 2) This method can achieve a more balanced performance in the event of an imbalance between classes. 3) The Bat-K-NN method is simpler and faster than the SVM and Random Forest methods, and can achieve better accuracy than the mentioned methods. 4) The Bat algorithm is an intelligent algorithm for large spaces. This allows the Bat-K-NN method to find optimal parameters when faced with complex data.

## 4. Dynamical analysis of the model

To analyze the dynamics of the proposed method in heterogeneous networks, the system equilibrium points and the basic fertility ratio can be calculated. In this section, these two factors are examined.

### 4.1. Equilibrium points

In this section, the dynamic behavior of the model is analyzed using the equilibrium point feature of the SEIRVS method. The equilibrium points are points that are free from any contamination and are a valuable tool for assessing the safety and continuity of the network using security attacks. For the equilibrium points of the proposed model, as in equation (20), we set all coefficients to zero.


$$\begin{array}{c} \frac{dS_{k}(t)}{dt}=\text{0},\;\frac{dE_{k}(t)}{dt}=\text{0},\;\frac{dI_{k}(t)}{dt}=\text{0},\;\frac{dR_{k}(t)}{dt}=\text{0},\frac{dV_{k}(t)}{dt}=\text{0} \end{array}$$ (20)

Assuming that the system is in equilibrium without attacks, in other words, Λ = μ and $S_{k}=I_{k}=\text{0}$, We obtain the equilibrium point for the malware-free state as follows:


$$\begin{array}{c} E_{0}=\left(\overset{0}{\underset{k}{S}},\;\overset{0}{\underset{k}{E}},\;\overset{0}{\underset{k}{I}},\;\overset{0}{\underset{k}{R}},\overset{0}{\underset{k}{V}}\right)=\left(1,\;0,\;0,\;0,0\right) \end{array}$$

All parameters $S_{k},E_{k},\;I_{k},R_{k},\;V_{k}$ are expressed in normalized form $S_{k}+E_{k}+\;I_{k}+\;V_{k}=\text{1}$. When $\overset{\text{0}}{\underset{k}{S}}=\text{1},$ all nodes are in a healthy (susceptible) state, and no one is infected, exposed, recovered, or vaccinated; in other words, the entire system is in a clean and safe state.

To calculate the native equilibrium point $E^{*}=(\overset{*}{\underset{k}{S}},\;\overset{*}{\underset{k}{E}},\;\overset{*}{\underset{k}{I}},\;\overset{*}{\underset{k}{R}},\;\overset{*}{\underset{k}{V}})$. We set the equations (1, 2, 3, 4, and 5) equal to zero:


$$\begin{array}{c} \left\{ \begin{array}{c} \Lambda +\beta R_{k}\;(t)+\tau V_{k}\;(t)-\lambda S_{k}\;(t)\theta -\mu S_{k}\;(t)=\text{0}\\ \lambda S_{k}(t)\theta -\epsilon E_{k}(\text{t})-\alpha E_{k}(\text{t})-\mu E_{k}(t)=\text{0}\\ \varepsilon E_{k}(t)-\sigma I_{k}(t)-\mu I_{k}(t)=\text{0}\\ \sigma I_{k}\;(t)-\gamma R_{k}(t)-\beta R_{k}\;(t)-\mu R_{k}\;(t)=\text{0}\\ \gamma R_{k}(\text{t})+\alpha E_{k}(\text{t})-\tau V_{k}(t)-\mu V_{k}(t)=\text{0} \end{array}\right.\; \end{array}$$

Obtaining the equilibrium point of this model is equation (21):


$$\begin{array}{c} \overset{*}{\underset{k}{E}}=-\frac{\Lambda }{A} \end{array}$$

$$\begin{array}{c} \overset{*}{\underset{k}{S}}=\frac{\left(\epsilon +\alpha +\mu \right)}{\lambda \theta }\overset{*}{\underset{k}{E}} \end{array}$$

$$\begin{array}{c} \overset{*}{\underset{k}{I}}=\frac{\epsilon }{\sigma +\mu }\overset{*}{\underset{k}{E}} \end{array}$$

$$\begin{array}{c} \overset{*}{\underset{k}{R}}=\frac{\sigma \varepsilon }{\left(\gamma +\beta +\mu )(\sigma +\mu \right)}\overset{*}{\underset{k}{E}} \end{array}$$

$$\begin{array}{c} \overset{*}{\underset{k}{V}}=\frac{\overset{*}{\underset{k}{E}}}{\tau +\mu }\left(\frac{\gamma \sigma \epsilon }{\left(\gamma +\beta +\mu )(\sigma +\mu \right)}+\alpha \right) \end{array}$$

Where,


$$\begin{array}{c} A=\frac{\beta \sigma \varepsilon }{\left(\gamma +\beta +\mu )(\sigma +\mu \right)}+\frac{\tau }{\tau +\mu }\left(\frac{\gamma \sigma \varepsilon }{\left(\gamma +\beta +\mu )(\sigma +\mu \right)}+\alpha \right)-(\epsilon +\alpha +\mu )-\frac{\mu (\epsilon +\alpha +\mu )}{\lambda \theta } \end{array}$$ (21)

### 4.2. Basic reproduction ratio (${𝐑}_{0}$)

The basic reproduction rate ($R_{\text{0}}$), in important mathematical concepts in epidemic models, is the average number of secondary infections from a primary infection during the life cycle of the infection. The basic R0 is a threshold parameter to represent the dynamic behavior of the model. The following conditions indicate the presence or absence of the disease:

1) If $R_{\text{0}}$>1. Each infected node can spread more than one new infection throughout the network; in other words, the spread of the epidemic is a botnet attack.
2) If $R_{\text{0}}$<1. The spread of the botnet attack has stopped, and an infection-free equilibrium has been established.

Here, the next generation method is used to calculate the basic reproduction ratio. This method is calculated using equation (22), where ρ(G) represents the spectral radius of the matrix G.


$$\begin{array}{c} R_{\text{0}}=\rho (FV^{-\text{1}}) \end{array}$$ (22)

Only the $E_{k}(t)\;$ and $I_{k}(t)$, states of the diffusion model are used to calculate $R_{\text{0}}$. First, the function F is calculated based on the infection rate in the groups $E_{k}(t)\;$ and $I_{k}(t)$, and the function V is calculated based on the transmission rate between these two groups. The matrix F and V is written in the relations (23) and (24).


$$\begin{array}{c} F=(\begin{array}{cc} \text{0} & \lambda \frac{<K^{\text{2}}>}{<K>}\\ \text{0} & \text{0} \end{array}) \end{array}$$ (23)

$$\begin{array}{c} V=(\begin{array}{cc} \epsilon +\alpha +\mu & \text{0}\\ -\epsilon & \sigma +\mu \end{array}) \end{array}$$ (24)

$$\begin{array}{c} V^{-\text{1}}=(\begin{array}{cc} \frac{\text{1}}{\left(\sigma +\mu \right)} & \text{0}\\ \frac{-\epsilon }{\left(\varepsilon +\alpha +\mu )(\sigma +\mu \right)} & \frac{\text{1}}{\left(\epsilon +\alpha +\mu \right)} \end{array}) \end{array}$$ (25)

$$\begin{array}{c} FV^{-\text{1}}=(\begin{array}{cc} \lambda \frac{<K^{\text{2}}>}{<K>}*\frac{-\epsilon }{\left(\varepsilon +\alpha +\mu )(\sigma +\mu \right)} & \lambda \frac{<K^{\text{2}}>}{<K>}*\frac{\text{1}}{\left(\epsilon +\alpha +\mu \right)}\\ \text{0} & \text{0} \end{array}) \end{array}$$ (26)

That $<K^{\text{2}}>\;=[\begin{array}{c} \text{1}\\ \text{2}\\ \vdots \\ \Delta \end{array}]\;[\begin{array}{cccc} \text{p} & \text{2p} & \ldots & \Delta \text{p} \end{array}]$ and $R_{\text{0}}$ is equal $\rho \left(FV^{-\text{1}}\right).\rho$ is spectral radius $FV^{-\text{1}}$

Now, the number of different configurations to prevent botnet attacks from spreading in the network is identified. So, the number of configurations that prevent attacks from spreading is as follows:


$$\begin{array}{c} R_{\text{0}}=\lambda \frac{<K^{\text{2}}>}{<K>}*\frac{-\epsilon }{\left(\varepsilon +\alpha +\mu )(\sigma +\mu \right)}\;\Rightarrow \;C_{critical}=\lambda \frac{<K^{\text{2}}>}{<K>}*\frac{-\epsilon }{\left(\varepsilon +\alpha +\mu )(\sigma +\mu \right)} \end{array}$$ (27)

## 5. Experiments and simulations

In this section, numerical simulations are performed to evaluate the dynamic performance of the Barabasi-Albert (BA)-based SEIRVS model in heterogeneous networks, as well as the combined GRO-BA-K-NN method for detecting botnet attacks using three datasets. In the proposed model, two types of security are considered, the first of which is performed using a recovery node to provide time for the second security. The second security layer includes an intrusion detection system for more advanced detection of botnet attacks. We implement the proposed method and all comparison algorithms in the Python programming language. We perform 100 independent runs of all methods on a personal computer with an Intel® Core™ i3 processor at 1.70 GHz and 4.0 GB of RAM running on Windows 10. Numpy, Pandas, Random, Math, Sklearn, matplotlib, and Time libraries were used for execution and conclusions. In the proposed epidemic model, the number of nodes was fixed during execution, and each node was in only one of the susceptible, exposed, infected, recovered, and vaccinated states.

### 5.1. Validation of SEIRVS Model

In this section, the SEIRVS model of different epidemic nodes is evaluated using other epidemic node models based on the standard framework of SEIR models, such as SEIRS, SIRS, SIRVS, classic SEIR and SEIVRS. In these models, additional components (vaccination or recovery) are introduced to capture different epidemic dynamics. These models are not explicitly defined in the relevant works. However, each of these models is briefly explained below.

- SIRS: The SIRS model is a simple model of disease spread that includes four states: susceptible individuals, infected individuals, and recovered individuals. In this model, immunity is not permanent, and individuals become susceptible again after a while. This model is suitable for diseases with short-term immunity.
- SEIRS: The SEIRS model is an extension of the SIRS model, with the addition of an at-risk phase, in which individuals are infected but not carriers. This model is suitable for diseases that have an incubation period, such as some viral diseases.
- SIRVS: The SIRVS model adds a vaccination scenario to the SIRS model. In this model, susceptible individuals can be vaccinated, although this immunity may not be permanent. In this model, vaccination policies are examined alongside temporary immunity.
- SEIR: The classic SEIR model is one of the most widely used classical epidemiology models suitable for diseases with stable immunity.
- SEIVRS: The SEIVRS model is a comprehensive model that has two modes: incubation period and vaccination, which, despite its high complexity, simulates a more realistic behavior of the disease.

**Fig 8:**
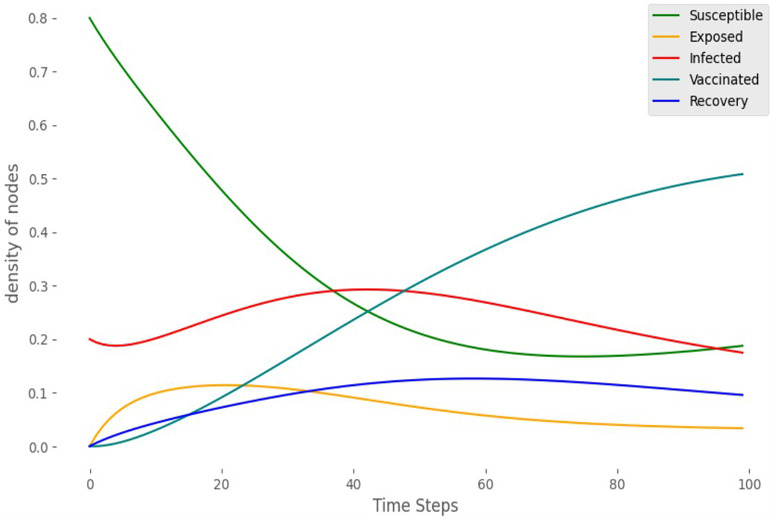
Dynamic behavior of the SEIRVS model.

Fig 8 shows the agent-based simulation of the spread of Button sentences based on the agent-based SEIRVS model in the BA model. In this simulation, 100 agents are randomly selected as infected nodes, and the rest are susceptible agents. In the BA network, the degree distribution function is a power law with thick tails. In this Fig, the overall trend of all cases under the parameters set in Table 4 is carried out without considering the diversity. Parameter estimation is a time-consuming and expensive challenge when there are many parameters. Here, the Monte Carlo method is used to estimate the parameters in Table 4. This method is a computational method based on random sampling that can handle complex problems well. This method does not solve a problem exactly, but simulates the average result using random values. The simulation results show that the density of infectious agents has increased significantly. According to this Fig, the density of infected nodes initially increases moderately, and with further expansion of attacks, they transmit the infection to their neighbors, and the density of infected agents increases. However, the density of susceptible agents decreases until they reach an equilibrium state. When the density of infected nodes reaches a peak, it starts to decrease over time and decrease until it reaches zero and the spread of attacks ends. The process of changes in the density of vulnerable nodes is the same as that of infected nodes, except that their rate of change is less than that of infected nodes. By reducing the density of nodes at risk and infected nodes, the density of vaccinated and recovered nodes is added, while during this process, the density of susceptible nodes is reduced.

**Table 4:** Value of parameters.

Symbol	Value	Symbol	Value
β	0.005	σ	0.03
τ	0.01	γ	0.06
λ	0.5	Λ	0
μ	0	α	0.03
ϵ	0.1		

**Fig 9:**
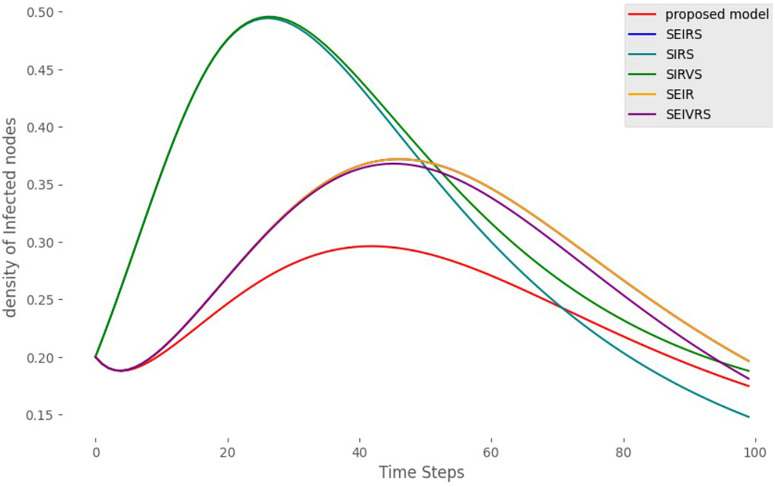
Compares the Infected node of the proposed model with other Infected node models.

Fig 9 compares the infected group in 5 models SIRS, SEIRS, SIRVS, SEIVRS, and the proposed model SEIRVS. All models are simulated using the same parameters. According to this Fig, the propagation process of botnet attacks of the proposed method has been reduced compared to other models. In the proposed model, due to the recovery and vaccination of vulnerable nodes, the propagation speed of attacks in the network has been reduced, and also the existence of a transition from the recovered and vaccinated state to the vulnerable state has caused the recovered and vaccinated nodes to become vulnerable again and spread the infection of botnet attacks. In general, the proposed model is more effective than epidemic models for simulating the propagation processes of attacks.

**Fig 10:**
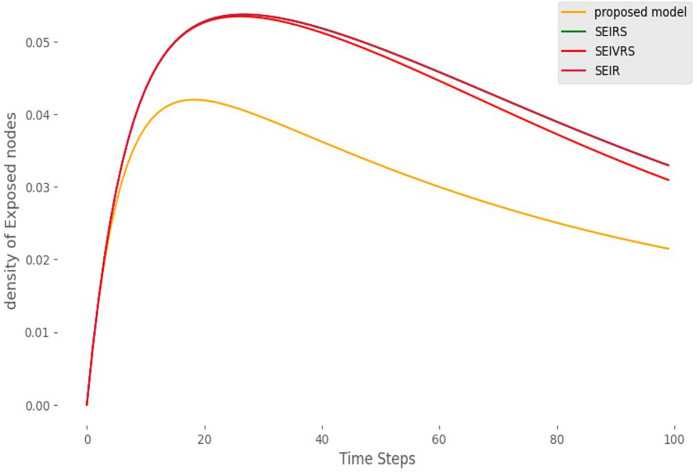
Compares the Exposed node of the proposed model with other Exposed node models.

In Fig 10, the diffusion density of nodes exposed to contamination initially increased significantly compared to other models because a path of susceptible, vaccinated, and recovered nodes entered it, but over time, it decreased significantly compared to other methods.

**Fig 11:**
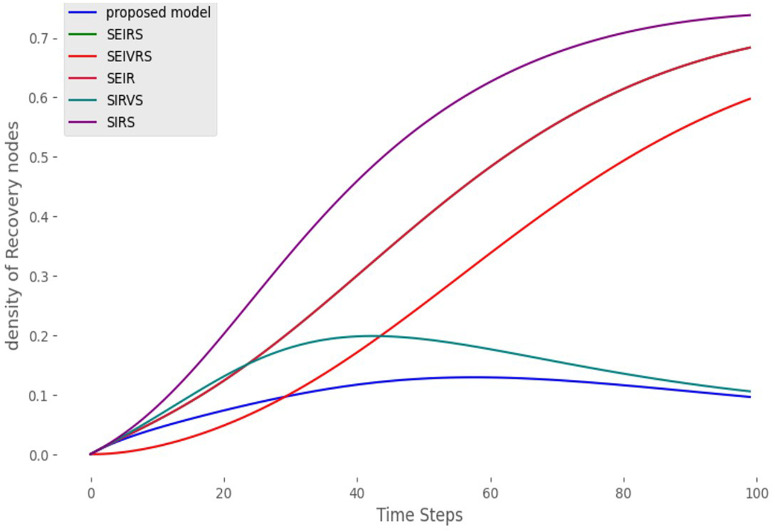
Compares the Recovery node of the proposed model with other Recovery node models.

**Fig 12:**
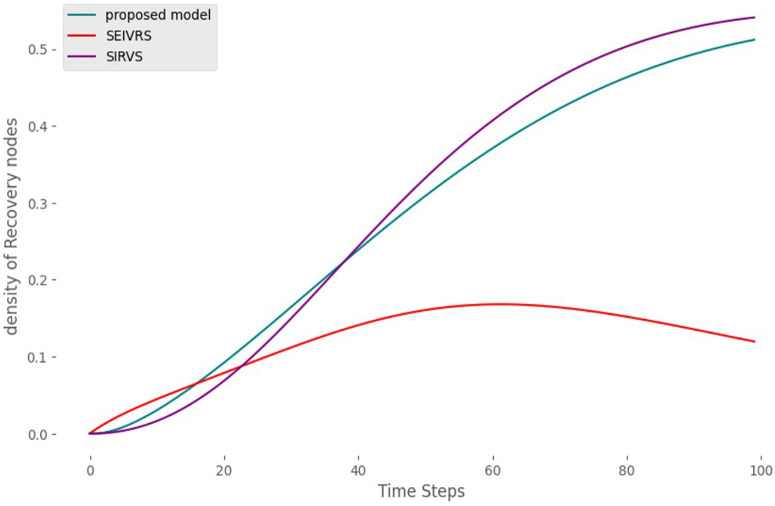
Compares the Vaccinated node of the proposed model with other Vaccinated node models.

Figs 11 and 12 examine the changes in the rank of the improved and vaccinated nodes in the proposed and other models. In the proposed model, the vaccination rate of infected nodes based on nodes with high rank increases the vaccination rate, which reduces the density of infected nodes, improves, and vaccinates nodes.

**Fig 13:**
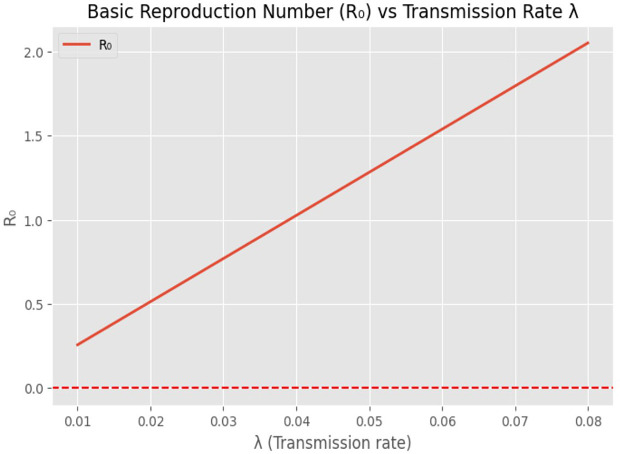
The impact of changes in λ on R₀.

The basic reproduction ratio (R_0_) is an epidemiological measure to describe the extent of the epidemic of an infectious disease in a network. If R_0_ is less than one, the epidemic in the network is reduced and eliminated, and if it is greater than one, each infected person infects more than one person and causes an epidemic by spreading the infection. This measure has a direct relationship with the parameters λ and ε and an inverse relationship with the parameters α and σ. In Fig 13, the values of R_0_ are analyzed for the increase in the parameter λ in the interval [0.01, 1]. According to this Fig, the value of R_0_ has increased with the increase in the infection rate.

**Fig 14:**
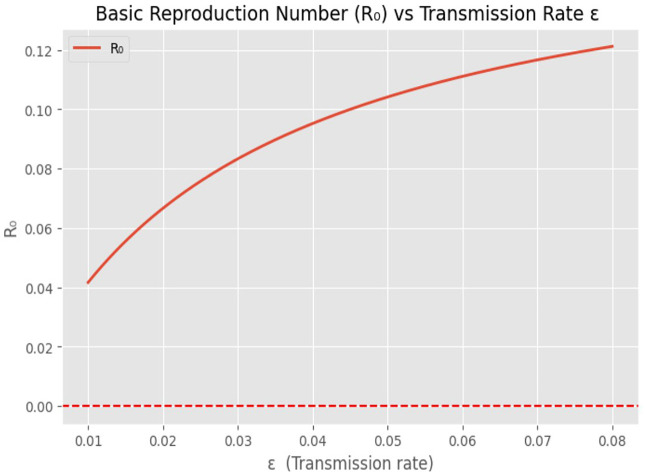
The impact of changes in ε on R₀.

In Fig 14, the relationship between R_0_ and the infection activation rate parameter in the interval [0.01, 0.5] is shown. According to this table and Fig, increasing the activation rate causes the disease spread to increase.

**Fig 15:**
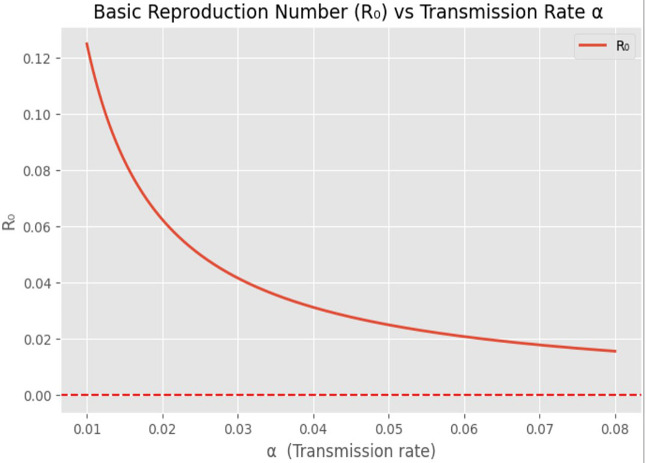
The impact of changes in α on R₀.

Fig 15 shows the rate of decrease in R_0_ values with increasing vaccination rate. According to this Fig, with an increasing vaccination rate, the rate of infection transmission and convergence decreases and can reach zero. This decreasing trend is analyzed in the graph of Fig 15 in the interval [0.01, 0.5].

**Fig 16:**
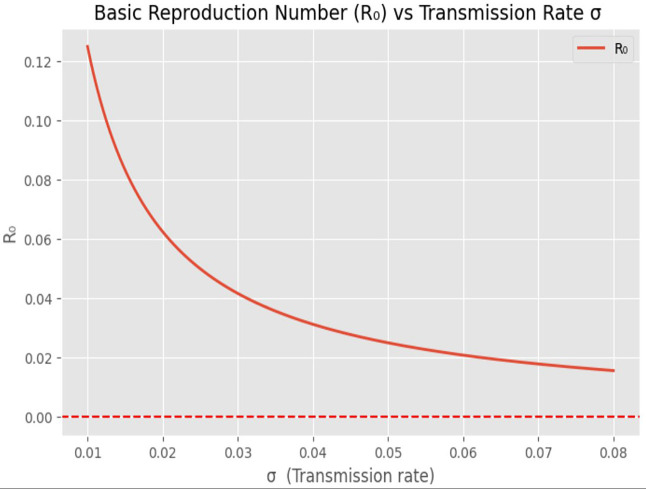
The impact of changes in σ on R₀.

Fig 16 shows the rate of decrease in R_0_ values with increasing recovery rate. According to this Fig, with increasing recovery rate, the rate of infection transmission and convergence decreases and can reach zero. This decreasing trend is analyzed in the graph of Fig 16 in the interval [0.1, 0.3].

### 5.2. Evaluation of the intrusion detection system

Here, an IDS combining machine learning and metaheuristic algorithms for detecting botnet attacks, named GRO-BA-K-NN, is presented. This method has been evaluated using three datasets: BOT-IOT, UNSW-NB15, and NLS-KDD, several single and combined methods, and other metrics such as accuracy, fitness, MSE, Precision, Recall, Specificity, F-measure, G-mean, MCC, and AUC. These performance metrics are evaluated based on four basic parameters: False Positive (FP) the number of positive samples that are incorrectly classified, False Negative (FN) the number of negative samples that are incorrectly classified as positive, True Positive (TP) the number of positive samples that are correctly classified as positive, and True Negative (TN) the number of negative samples that are correctly classified as negative. The results obtained are the average of 100 independent runs of the algorithm. In addition, to show the stability of the algorithm, the standard deviation (SD) and the standard error of the mean (SE) have also been calculated. The standard error has been calculated using equation (28). The 95% confidence interval for the mean of each criterion has been obtained using SE and is given by equation (29).


$$\begin{array}{c} \frac{SD}{\sqrt{\text{1}\text{0}\text{0}}}=SE \end{array}$$ (28)

$$\begin{array}{c} SE*\text{1}.\text{9}\text{6}\pm \overset{―}{X}={CI}_{\text{9}\text{5}\%} \end{array}$$ (29)

Below, brief explanations about the datasets used are provided.

- BOT-IOT: It is a dataset consisting of Internet of Things (IoT) device traffic and is suitable for intrusion detection and botnet attacks. In this dataset, each record represents a network flow or traffic sample, and the features of this dataset include numerical features as well as protocol and service features. It contains over 72 million records from hundreds of networks and includes attacks such as DoS (Denial of Service), DDoS (Distributed Denial of Service), OS Scan, Service Scan, Data Exfiltration, and Keylogging [38].
- UNSW-NB15: This dataset consists of 2,540,044 records and was generated by the Australian Centre for Cyber Security (ACCS) at the University of New South Wales. The dataset contains real and simulated examples of network traffic. The dataset includes 49 features and nine traffic types such as Normal, DoS (Denial of Service), Probe, R2L (Remote to Local), U2R (User to Root), Exploits, General, Web Attack, and Intrusion [38].
- NLS-KDD: This dataset is an improved version of the KDD Cup 1999 dataset used for intrusion detection attacks. This dataset contains 49,000 records with 43 features. This dataset is suitable for the field of network security and attack detection. This dataset includes practical attacks such as DoS (Denial of Service), Probe Attack, R2L (Remote to Local) operations and U2R (User to Root) applications [39].

#### 5.2.1. The effect of the proposed feature selection method.

In this section, the proposed method for feature selection is evaluated using accuracy and fitness value, and several other algorithms that are explained below.

Differential Evolution (DE): This algorithm is based on the evolutionary behavior of the population. In each generation, the solutions are passed to the next generation using the process of combination, mutation, and selection. This method can find the best solution for complex and unknown objective functions. The main difference of this algorithm from other evolutionary algorithms is in the mutation process, which uses the differences between the population members [40].

Whale Optimization Algorithm (WOA): The Whale Optimization Algorithm (WOA) is based on the hunting behavior of the humpback whale, which creates bubbles by spinning around prey, which it uses to trap the prey. The main behaviors of this algorithm include random search, prey encirclement, and spiral attack [41].

Gray Wolf Optimization (GWO): The Gray Wolf Optimizer (GWO) algorithm is a swarm intelligence algorithm based on the social and hunting behavior of gray wolves, which has a hierarchy of α, β, δ, and ω for leader selection. This algorithm consists of three phases: prey encirclement, hunting, and attack. This algorithm is very fast and resistant to local optima [42].

Particle Swarm Optimization (PSO): The Particle Swarm Optimization (PSO) algorithm is based on the social behavior of birds in a flock that use their own and others’ experience to search for food, and its main feature is to create a balance between group and individual experience. This algorithm has a low number of parameters and a high convergence speed [43].

Firefly Algorithm (FA): The Firefly Algorithm (FA) is a swarm intelligence algorithm based on the behavior and glow of fireflies. In this algorithm, each firefly represents a solution, and its brightness is proportional to the value of the fitness function. The less luminous firefly moves towards the more luminous one. This algorithm is resistant to local optima [44].

**Fig 17:**
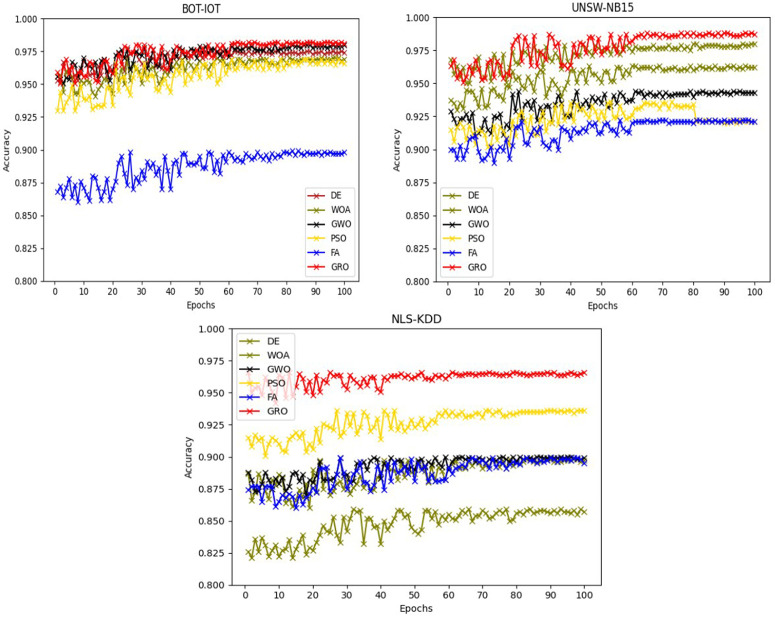
The accuracy of the feature selection curve of the GRO method compared to another algorithm.


Accuracy is one of the most common metrics for evaluating the performance of machine learning and metaheuristic algorithms. This metric represents the percentage of correct predictions out of all predictions. This metric is calculated using equation (28). In Table 6, the accuracy value of the proposed method in feature selection is compared with other methos include Differential Evolution (DE) [35], Whale Optimization Algorithm (WOA) [28], Gray Wolf Optimization (GWO) [36], Particle Swarm Optimization (PSO) [30], Firefly Algorithm (FA) [33]. This Table shows the average accuracy of the proposed model along with the standard deviation and the 95% confidence interval. The average accuracy reflects the overall performance of the model across multiple test runs, while the standard deviation indicates the dispersion and stability of the results, with a lower standard deviation signifying greater model stability. The 95% confidence interval is also included, which indicates the reliability of the obtained results. According to this Table, the proposed method has selected a better subset of features with values (0.982, 0.988, and 0.966) than other methods. In Table 5, the training dataset was trained by the proposed method and the mentioned methods, and then all methods were classified using the nearest neighbor algorithm, and their performance accuracy was evaluated. To create the same conditions for all models for comparison, a fixed and identical experimental setup was used. In all datasets, a fixed ratio of 30%−70% of the dataset was randomly divided into training and testing subsets, and the classifier with a fixed value of k and the distance criterion in the k-NN algorithm were the same in all experiments. In Fig 17, the accuracy curve is shown in 100 iterations. The figures show the convergence speed of the methods to the desired solution. Based on these figures, three important aspects can be concluded: 1) convergence rate, 2) final accuracy, and 3) stability.

**Table 5:** Feature selection accuracy of GRO compared with other algorithms.

Method	BOT-IOT (SD±$\overset{―}{𝐗}$)	CI 95%	UNSW-NB15 (SD±$\overset{―}{𝐗}$)	CI 95%	NLS-KDD (SD±$\overset{―}{𝐗}$)	CI 95%
DE	0.975 ± 0.005	[0.965, 0.985]	0.980 ± 0.004	[0.972, 0.988]	0.899 ± 0.010	[0.879, 0.919]
WOA	0.970 ± 0.006	[0.958, 0.982]	0.963 ± 0.007	[0.949, 0.977]	0.859 ± 0.012	[0.835, 0.883]
GWO	0.980 ± 0.004	[0.972, 0.988]	0.945 ± 0.008	[0.929, 0.961]	0.900 ± 0.010	[0.880, 0.920]
PSO	0.969 ± 0.007	[0.955, 0.983]	0.936 ± 0.009	[0.918, 0.954]	0.936 ± 0.010	[0.916, 0.956]
FA	0.899 ± 0.010	[0.879, 0.919]	0.922 ± 0.009	[0.904, 0.940]	0.899 ± 0.011	[0.877, 0.921]
GRO	0.982 ± 0.003	[0.976, 0.988]	0.988 ± 0.002	[0.984, 0.992]	0.966 ± 0.007	[0.952, 0.980]

**Table 6:** Comparison Min fitness of compared algorithms.

Method	BOT-IOT	UNSW-NB15	NLS-KDD
DE	0.03	0.02	0.182
WOA	0.029	0.029	0.362
GWO	0.02	0.1	0.172
PSO	0.03	0.110	0.109
FA	0.16	0.121	0.184
GRO	0.022	0.017	0.033

$$\begin{array}{c} \text{Accuracy}=\frac{\text{TP}+TN}{TP+\text{FP}+\text{TN}+\text{FN}} \end{array}$$ (28)

To evaluate the proposed method in feature selection, the fitness criterion is used. This criterion is an evaluation criterion to evaluate the performance of a method to show the optimality of that method. The lower its value, the better the subset of selected features. Table 6, shows the minimum fitness value over 100 iterations. According to this Table, the proposed method with fitness values (0.022, 0.017, and 0.033) has selected a better subset of features.

#### 5.2.2. The effect of the proposed detection attack method.

In this section, the proposed method for detecting attacks is presented with several other evaluation criteria that are briefly explained below.

- BA-DE: This method is based on a combination of two methods, Bat algorithm (BA) and Differential Evolution (DE). In this method, feature selection is first performed using the BA algorithm, then training is performed using the DE algorithm, and finally, attack detection is performed using the decision tree algorithm.
- PCA-BA: This method is based on a combination of the Bat algorithm (BA) and Principal Component Analysis (PCA) algorithms. First, feature selection is performed using the PCA algorithm, second, training is performed using the BA algorithm, and finally, attack detection is performed using the decision tree algorithm.
- MQBHOA: This method is a combination of the Horse Herd Optimization Algorithm (HOA) with quantum computing concepts in a multi-objective manner. In this method, first, the behaviors of the horses in the herd are used to select features, which are converted into a discrete algorithm using the floor function, then optimized using quantum computing concepts, and finally, attacks are identified using the decision tree algorithm.
- FA-K-Means: This method is a combination of the Firefly Algorithm (FA) and K-Means algorithms. In this method, first feature selection is performed using the Firefly Algorithm (FA), then attack detection is performed using the K-Means algorithm.
- WOA-BA-K-NN: This method is a combination of the whale optimization algorithm (WOA) and the Bat algorithm (BA). First, feature selection is performed using the whale optimization algorithm (WOA), second, training is performed using the Bat algorithm (BA), and in the final stage, attack detection is performed using the nearest neighbor algorithm.
- GWO-PSO-K-Means: This method is a combination of Gray Wolf Optimization (GWO) and Particle Swarm Optimization (PSO) algorithms. First, the feature selection process is performed using the GWO algorithm, then the Particle swarm optimization (PSO) algorithm, and finally, the attack detection is performed using the K-Means algorithm.
- FCM-SWA: The FCM-SWA method combines the fuzzy C-mean (FCM) algorithm with a sperm whale algorithm (SWA). In this method, the SWA optimization algorithm is used to increase the performance of the FCM and provide effective defense against attacks.
- HHO‐SCA: The HHO-SCA method is based on the Sine-Cosine (SCA) algorithm and the Harris-Hawks (HHO) algorithm. Using this method, the parameters of the SVM algorithm are optimized and the SVM algorithm is used to classify attacks.
- LOFSGWO: The LOFSGWO method is based on the Lion Optimization Algorithm (LOA) and, in particular, the Gray Wolf Optimizer (GWO). In this method, the Lion Optimization Algorithm (LOFS) and Gray Wolf GWO are used to reduce hazardous parameters.
- BGWO-EESNN: A hybrid method based on deep learning algorithms was proposed. In this method, feature selection is performed using Binary Grey Wolf Optimization (BGWO), and an Elemental Enhanced Spiking Neural Network (EESNN) is used for attack detection.

**Fig 18:**
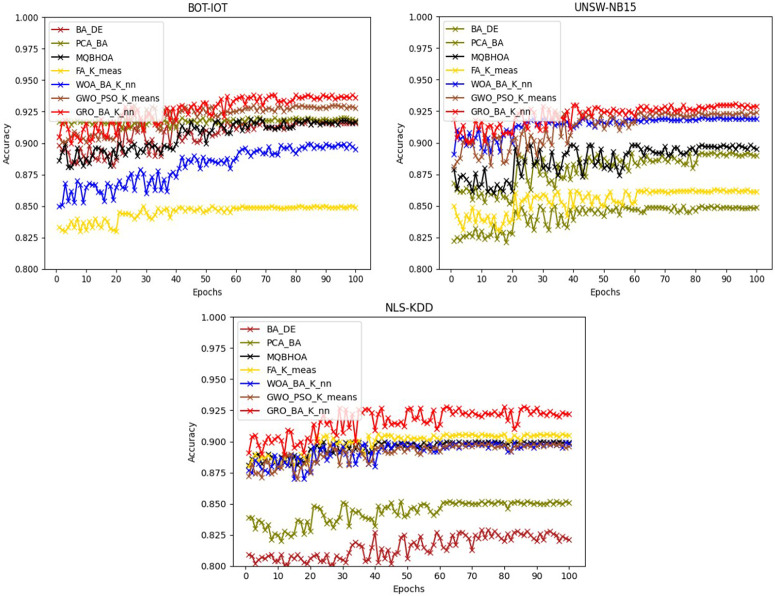
The accuracy curve of the GRO-BA-K-NN method compared to another algorithm.

Accuracy is an evaluation criterion for evaluating the performance of a prediction method that determines the number of correct predictions out of all predictions, which is suitable for a balanced data set. If the performance is unbalanced, it will have a negative impact. This criterion is calculated using equation (28). According to Table 7, the proposed method with values (0.938, 0.931, and 0.928) has been able to predict more correct cases better than other methods incudes Bat algorithm (BA) and Differential Evolution (DE) (BA-DE) [31], Bat algorithm (BA) and Principal Component Analysis (PCA) (BA-PCA) [27], MQBHOA [25], Firefly Algorithm (FA) and K-Means algorithms (FA-K-Means) [3], whale optimization algorithm (WOA) and the Bat algorithm (BA)(WOA-BA-K-NN) [32], Gray Wolf Optimization (GWO) and Particle Swarm Optimization (PSO) (GWO-PSO-K-Means) [26], fuzzy C-mean (FCM) algorithm with a sperm whale algorithm (SWA) (FCM-SWA) [17], Sine-Cosine (SCA) algorithm and the Harris-Hawks (HHO) (HHO‐SCA) [29], Lion Optimization Algorithm (LOA) and, in particular, the Gray Wolf Optimizer (GWO) (LOFSGWO) [24], Binary Gray Wolf Optimization (BGWO) Element Enhanced Spike Neural Network (EESNN) (BGWO-EESNN) [16]. According to this Table, the proposed method has achieved the highest average accuracy with the lowest variance (0.938, 0.931, and 0.928). In Fig 18, the accuracy curve is shown in 100 iterations.

**Table 7:** Classification accuracy of GRO-BA-K-NN compared with other algorithms.

Method	BOT-IOT (SD±$\overset{―}{𝐗}$)	CI 95%	UNSW-NB15 (SD±$\overset{―}{𝐗}$)	CI 95%	NLS-KDD (SD±$\overset{―}{𝐗}$)	CI 95%
BA-DE	0.918 ± 0.274	[0.864, 0.972]	0.892 ± 0.311	[0.831, 0.953]	0.829 ± 0.377	[0.755, 0.903]
PCA-BA	0.920 ± 0.272	[0.867, 0.973]	0.850 ± 0.357	[0.780, 0.920]	0.852 ± 0.355	[0.782, 0.922]
MQBHOA	0.919 ± 0.273	[0.866, 0.972]	0.899 ± 0.300	[0.840, 0.958]	0.900 ± 0.300	[0.841, 0.959]
FA-K-Means	0.850 ± 0.357	[0.780, 0.920]	0.863 ± 0.343	[0.796, 0.930]	0.906 ± 0.292	[0.849, 0.963]
WOA-BA-K-NN	0.899 ± 0.300	[0.840, 0.958]	0.920 ± 0.272	[0.867, 0.973]	0.899 ± 0.300	[0.840, 0.958]
GWO-PSO-K-Means	0.930 ± 0.255	[0.880, 0.980]	0.924 ± 0.265	[0.872, 0.976]	0.898 ± 0.302	[0.839, 0.957]
FCM-SWA	0.928 ± 0.258	[0.878, 0.978]	0.848 ± 0.358	[0.778, 0.918]	0.850 ± 0.357	[0.780, 0.920]
HHO‐SCA	0.929 ± 0.257	[0.879, 0.979]	0.850 ± 0.357	[0.780, 0.920]	0.896 ± 0.305	[0.836, 0.956]
LOFSGWO	0.930 ± 0.255	[0.880, 0.980]	0.920 ± 0.272	[0.867, 0.973]	0.908 ± 0.289	[0.851, 0.965]
BGWO-EESNN	0.929 ± 0.257	[0.879, 0.979]	0.864 ± 0.343	[0.797, 0.931]	0.908 ± 0.289	[0.851, 0.965]
GRO-BA-K-NN	0.938 ± 0.241	[0.891, 0.985]	0.931 ± 0.254	[0.881, 0.981]	0.928 ± 0.258	[0.878, 0.978]

The MSE (Mean Squared Error) metric measures the mean squared difference between the predicted values and the actual values. It shows the amount of deviation of the predicted data points from the actual data points. The lower it is, the better the method performed. This metric is calculated using equation (29). According to Table 8, the proposed method with values (0.061, 0.068, and 0.1) had a prediction close to the actual classes compared to other methods.

**Table 8:** Compare the error of the GRO-BA-K-NN method.

Method	BOT-IOT	UNSW-NB15	NLS-KDD
BA-DE	0.15	0.17	0.3
PCA_BA	0.134	0.215	0.21
MQBHOA	0.142	0.165	0.152
FA-K-Means	0.210	0.236	0.143
WOA-BA-K-NN	0.163	0.133	0.155
GWO-PSO-K-Means	0.08	0.126	0.16
FCM-SWA	0.09	0.22	0.215
HHO‐SCA	0.093	0.213	0.169
LOFSGWO	0.0.9	0.134	0.138
BGWO-EESNN	0.1	0.238	0.130
GRO-BA-K-NN	0.061	0.068	0.1


$$\begin{array}{c} MSE\;=\frac{\text{1}}{n}\sum_{i}{\left(xi-\;yi\right)}^{\text{2}} \end{array}$$ (29)

In Tables 9, 10 and 11, the proposed method is evaluated using important metrics such as Precision, Recall, F-Measure, Specificity, and G-Measure. The Decision metric includes the positive samples that are correctly predicted, and the higher it is, the lower the false positive rate will be. The Recall metric shows the rate of true positives predicted by the model; the higher it is, the lower the number of false negatives. The F-Measure strikes a balance between Precision and Recall. The Specificity metric includes the rate of correct identification of negatives. G-Measure: The average between sensitivity and specificity that can handle unbalanced datasets well. These metrics are calculated using the following equations. According to these Tables, the proposed method with values of (0.953, 0.938, and 0.962) in the F-criterion has been able to reduce the false positive and negative rates and increase the true negative and positive rates. Specifically, in Table 10, the MQBHOA algorithm has obtained a Specificity value of 0.889 on the BOT-IOT dataset, which indicates that it has a high ability to reduce the false positive rate and increase the overall accuracy of detecting attacks. This value indicates that the proposed method and the MQBHOA algorithm have been able to minimize the false positive rate and have a significant performance in correctly classifying negative samples.

**Table 9:** Comparison of the Confusion Matrix values in BOT-IOT.

Method	Precision	Recall	F-measure	specificity	G-mean
BA-DE	0.91	0.953	0.931	0.799	0.872
PCA_BA	0.915	0.969	0.941	0.9	0.933
MQBHOA	0.914	0.949	0.931	0.889	0.918
FA-K-Means	0.846	0.9	0.872	0.785	0.840
WOA-BA-K-NN	0.893	0.910	0.901	0.850	0.879
GWO-PSO-K-Means	0.923	0.959	0.940	0.862	0.909
FCM-SWA	0.920	0.950	0.934	0.958	0.953
HHO‐SCA	0.922	0.958	0.939	0.962	0.959
LOFSGWO	0.930	0.963	0.946	0.960	0.961
BGWO-EESNN	0.899	0.936	0.917	0.787	0.858
GRO-BA-K-NN	0.934	0.972	0.953	0.878	0.953

**Table 10:** Comparison of Confusion Matrix values in UNSW-NB15.

Method	Precision	Recall	F-measure	specificity	G-mean
BA-DE	0.9	0.861	0.880	0.909	0.884
PCA_BA	0.862	0.809	0.834	0.799	0.803
MQBHOA	0.9	0.869	0.884	0.9	0.884
FA-K-Means	0.87	0.809	0.838	0.79	0.799
WOA-BA-K-NN	0.935	0.89	0.911	0.886	0.887
GWO-PSO-K-Means	0.910	0.909	0.909	0.899	0.903
FCM-SWA	0.860	0.858	0.858	0.809	0.833
HHO‐SCA	0.799	0.785	0.791	0.899	0.840
LOFSGWO	0.912	0.910	0.910	0.9	0.904
BGWO-EESNN	0.87	0.810	0.838	0.78	0.794
GRO-BA-K-NN	0.938	0.938	0.938	0.921	0.938

**Table 11:** Comparison of Confusion Matrix values in NLS-KDD.

Method	Precision	Recall	F-measure	specificity	G-mean
BA-DE	0.819	0.83	0.824	0.774	0.785
PCA_BA	0.850	0.836	0.842	0.86	0.847
MQBHOA	0.915	0.893	0.903	0.869	0.880
FA-K-Means	0.910	0.896	0.902	0.889	0.892
WOA-BA-K-NN	0.9	0.869	0.884	0.880	0.874
GWO-PSO-K-Means	0.9	0.896	0.897	0.856	0.875
FCM-SWA	0.852	0.809	0.829	0.799	0.803
HHO‐SCA	0.895	0.881	0.887	0.839	0.859
LOFSGWO	0.909	0.9	0.904	0.899	0.899
BGWO-EESNN	0.909	0.897	0.901	0.887	0.891
GRO-BA-K-NN	0.928	1	0.962	0.899	0.963


$$\begin{array}{c} Precistion=\frac{TP}{TP+FP} \end{array}$$ (30)

$$\begin{array}{c} Recall\;=\frac{TP}{TP+FN} \end{array}$$ (31)

$$\begin{array}{c} Specificity=\frac{TN}{TN+FP} \end{array}$$ (32)

$$\begin{array}{c} F-measure=\text{2}*\frac{\left(precision*recall\right)}{precision+recall} \end{array}$$ (33)

$$\begin{array}{c} \text{G}-\text{mean}=\sqrt{Recall*specificity} \end{array}$$ (34)

**Fig 19:**
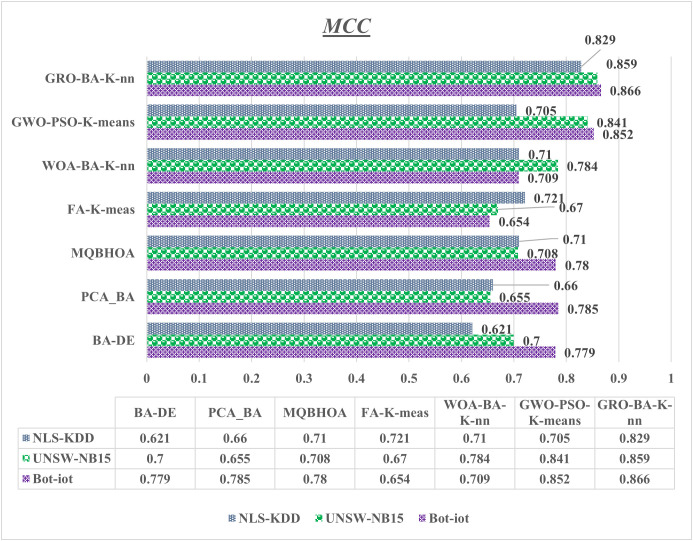
Compare the proposed method with the MCC criterion value.


MCC (Matthews Correlation Coefficient) is a comprehensive performance measure that is evaluated using the parameters of true positives (TP), false positives (FP), true negatives (TN) and false negatives (FN). This measure can handle unbalanced data sets well and is a value between −1 and +1. If MCC is equal to +1, it means perfect prediction; if it is equal to 0, it means random prediction, and if it is equal to −1, it means reverse prediction. This measure is calculated using equation (35). In Fig 19, the performance of the proposed method with other methods has been investigated. According to this Fig, the proposed method with values (0.829,0.859,0.866) can make predictions more completely than other methods.

$$\begin{array}{c} MCC=\frac{\left(TP*TN)-(FP*FN\right)}{\sqrt{\left(TP+FP)(TP+FN)(FN+FP)(TN+FN\right)}} \end{array}$$ (35)

In Table 12, the proposed method is evaluated with other methods using the training time criterion. These criteria are in seconds. According to this Table, the proposed method was able to reduce the execution time by times of 320, 331, and 325.

**Table 12:** Compare the proposed method with the training time criterion value.

Method	BOT-IOT	UNSW-NB15	NLS-KDD
BA-DE	365	451	398
PCA_BA	384	470	420
MQBHOA	350	418	370
FA-K-Means	290	299	290
WOA-BA-K-NN	462	480	465
GWO-PSO-K-Means	400	421	412
FCM-SWA	356	498	396
HHO‐SCA	468	452	400
LOFSGWO	400	421	405
BGWO-EESNN	365	418	368
GRO-BA-K-NN	320	331	325

**Fig 20:**
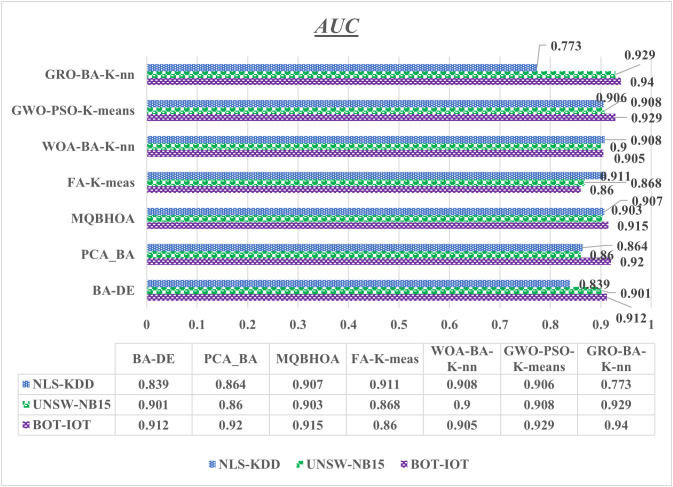
Compare the proposed method with the AUC criterion value.

Area Under the Curve (AUC) is a numerical measure used to evaluate the performance of detection methods. This measure is between 0 and 1. If AUC = 0.5, the method has a random performance and is not able to make any distinction. If AUC is close to 1, the method has a high ability to make distinctions. If AUC is close to 0, the method has performed inversely. This measure can measure the performance of the model to make distinctions using a comprehensive view across all thresholds. This measure can also handle unbalanced data better than other measures and will still have stable performance if there are changes in the data distribution. In Fig 20, the performance of the proposed method using this measure is compared with other methods. According to this Fig, the proposed method has performed better with values of (0.773, 0.929, and 0.94). According to Fig 20, the proposed method has the best performance in all datasets. In the NLS-KDD dataset, the performance of the proposed method was weaker than some of the comparative methods due to reasons such as unbalanced class distribution, different traffic patterns, and sensitivity of the AUC criterion to changes in the false positive rate. However, this method had competitive and stable performance in other datasets and criteria, which indicates the overall capability of the model in detecting attacks.

In Table 13, the performance of different methods in detecting types of network attacks has been evaluated using the BOT-IOT dataset. In this Table, each row corresponds to a method, and the performance of that method for each type of attack is given below it. The attacks examined include Normal, DOS (Denial of Service), DDoS (Distributed Denial of Service), Reconnaissance, Theft attacks. In this Table, the average Precision and average Recall criteria have been used for comparison. According to this Table, the GRO-BA-KNN method, with the highest Precision and Recall values, has performed very well in detecting types of attacks. This indicates that it is the ideal method for an IDS with the lowest error.

**Table 13:** Comparison of methods in terms of Precision and Recall for each attack class.

Algorithms	Class attack	Class Precision	Class Recall
BA-DE	Normal	0.91	0.9
	DOS	0.842	0.835
	DDOS	0.895	0.821
	Reconnance	0.936	1
	Theft	0.835	0.965
PCA-BA	Normal	0.9	0.892
	DOS	0.835	0.74
	DDOS	0.765	0.8
	Reconnance	0.9	0.936
	Theft	1	0.874
MQBHOA	Normal	0.8	0.864
	DOS	0.795	0.8
	DDOS	0.936	0.915
	Reconnance	0.909	0.9
	Theft	0.847	0.869
FA-K-Means	Normal	0.564	0.587
	DOS	0.6	0.608
	DDOS	0.8	0.763
	Reconnance	0.695	0.7
WOA-BA-K-NN	Theft	0.9	0.89
	Normal	0.847	0.894
	DOS	0.9	0.754
	DDOS	0.864	0.9
	Reconnance	0.918	0.899
	Theft	0.9	0.914
GWO-PSO-K-Means	Normal	0.926	0.964
	DOS	0.921	0.918
	DDOS	0.8	0.948
	Reconnance	0.863	0.918
	Theft	0.9	0.874
GRO-BA-K-NN	Normal	1	0.969
	DOS	0.95	0.948
	DDOS	1	0.96
	Reconnance	0.974	0.936
	Theft	0.97	1

## 6. Discussion

In the proposed intrusion detection system, the golden ratio optimization algorithm is used for feature selection, the bat optimization algorithm is used for training, and the K-NN algorithm is used for attack classification. In this method, the golden ratio optimization algorithm has an optimal performance that is neither unstable nor stuck in a local minimum, resulting in faster convergence and higher stability for feature selection. In many metaheuristic algorithms, the choice of learning rate or update factor is random. The golden ratio is an optimal ratio that increases accuracy and reduces overfitting. In this method, BA is used as an optimization mechanism to improve the detection performance of the K-nearest neighbor classifier. Here, each bat (a set of selected features) in the population is a solution, the position of each bat is a subset of the features, and the speed of the bat towards better solutions in the search space is based on the fitness value. The fitness function is the detection accuracy of the KNN, which is updated during the process of bats’ speed and position. By obtaining the optimal solution, the KNN algorithm is trained with the optimized parameters, and is used to detect attacks. Despite the good results obtained from the proposed GRO-BA-K-NN method, this method can increase the number of iterations due to simultaneous optimization of parameters and calculate the distances in K-NN repeatedly, which will be very time-consuming in large data, resulting in increased computational cost. In the meta-heuristic algorithms BA and GRO, the initial setting of parameters is very important. If the parameters are set incorrectly, this method gets stuck in local minima and does not achieve optimal results. In unbalanced data that has a minority, the K-NN algorithm assigns the samples to the minority class. The GRO-BA-K-NN method is optimized specifically for each data set, and when faced with a new data set, it has poor performance and suffers from overfitting. Epidemic modeling of botnet attack spread is used to simulate and predict the spread of botnet attacks in network security, but IDS is designed to prevent botnet attacks. This epidemic model helps to analyze the epidemic patterns of botnet attacks, and IDS increases the detection speed and prevents the spread of attacks. The more accurate the epidemic model, the higher the detection accuracy of the IDS and the lower its speed. The simpler the model, the lower the detection accuracy, but the higher the detection speed. The more accurate the epidemic model, the more concentrated the required resources of the IDS and identifies critical points in the network, resulting in faster and more effective response. For transaction management, a relatively simple model is designed here and the intelligent GRO-BA-KNN algorithms are used for detection, which reduces the processing cost of the IDS by selecting the dimension feature. The proposed method prevents the spread of botnet attacks by timely detecting them. In the GRO-BA-KNN hybrid method, the K-NN algorithm is a lazy learning algorithm that has no explicit training step but has a high computational cost. This method requires setting the parameters of the number of neighbors (k) and the distance criterion. The computational cost of the GRO algorithm is O(T × N × D), where the parameter T represents the number of iterations, the parameter N represents the population size, and the parameter D represents the number of variables. The computational cost of the BA algorithm is O(T*N(D + C)), where the parameter C represents the cost of the objective function. The computational cost of the K-NN algorithm is O(1) during training and O(Q*(M*D+MlogM)) during prediction, where each distance in the D dimension is O(D), the total distances are O(M*D), and the full sorting is O(MlogM), and the selection of the smallest K is O(M). The computational cost of evaluating each candidate using the GRO-BA-KNN method is $\text{O}(T_{g}*N_{g}*\text{D}+\;T_{b}*N_{b}*\text{D}+(N_{g}*T_{g}+N_{b}*T_{b})*\text{C})$. In the BA–KNN method, it has a high cost due to parameter search, and in the GRO–BA–KNN method, the algorithm converges faster, and the stability of convergence improves by reducing the number of iterations.

## 7. Conclusions and future works

In this paper, a pan-epidemic model for the propagation of botnet attacks called SEIRVS in heterogeneous networks is investigated. The topology of the proposed model is based on the power-degree distribution using the Barabasi-Albert model. In this model, two recovery and vaccination nodes are used to eliminate the infection. In the first node, the infection is temporarily eliminated, and in the second node, vaccination is performed, and the propagation process is slowed down for a longer period. Here, numerical simulations and the effects of various parameters are performed to verify the analytical results. In addition, we have investigated the model. The simulation results showed that the dynamics of the model are completely affected by the basic reproduction ratio and the number of diverse software packages. Here, an intrusion detection method for detecting botnet attacks called GRO-BA-K-NN, based on the combination of hyper-processing and machine learning algorithms, is also presented. This method consists of 3 steps: first, using preprocessing of the cleaned dataset; second, using the GRO algorithm, the subset of the best features with the lowest fitness value; third, training using the BA algorithm and classifying normal and malicious traffic using the K-NN algorithm. This method has been evaluated using three datasets: BOT-IOT, UNSW-NB15, NLS-KDD, and several combined and single algorithms. The evaluation results show that the proposed SEIRVS epidemic model does not spread when the R0 value is less than one, and when the R_0_ value is greater than one, the spread of infection in the network increases. This model has reduced the spread of infection and has performed better than other methods. This model reduced the spread of infection and performed better than other methods. The simulation results of the proposed IDS show that the proposed method was able to reduce the false negative and false positive rates and identify attacks with accuracy (0.928, 0.934, 0.928), recall (0.972, 0.938, 1), F-measure (0.953, 0.938, 0.962), AUC (0.773, 0.929, 0.940) with the values obtained in the criteria.Operators can use the proposed model to simulate and predict the spread of botnet attacks in the network, which helps to design targeted defense measures. The operator collects data to estimate the parameters of the model and can model the current state of the network. With the help of this model, operators can estimate the level of infection of the network in the future, identify critical points, examine the impact of factors such as increasing the vaccination rate and disconnecting infected nodes, help reduce the cost of emergency response, and make smart decisions in real time with the help of this model. IDS integration is the combination of different IDS sources and types to increase the efficiency of threat detection. In the real world, this can significantly improve performance. Individual IDSs can generate a large number of false alarms, and each of them can detect only a small part of the threats on its own, but with integration, 1) the system can compare alarms, reduce the number of false alarms, and increase the accuracy of real detection. 2) The system uses a wider variety of data to train detection models that can be resistant to new threats.

In future work, we plan to: 1) study new virus strains that can escape from different immune systems. 2) Building a model to increase immunity after infection or vaccination, to reduce possible re-sensitization 3) Using deep learning algorithms that are capable of recognizing complex patterns. 4) Increasing the processing speed of the method to detect and respond to attacks in real time.
